# Remission and Relapse of Dyslipidemia After Vertical Sleeve Gastrectomy vs Roux-en-Y Gastric Bypass in a Racially and Ethnically Diverse Population

**DOI:** 10.1001/jamanetworkopen.2022.33843

**Published:** 2022-09-28

**Authors:** Karen J. Coleman, Anirban Basu, Lee J. Barton, Heidi Fischer, David E. Arterburn, Douglas Barthold, Anita Courcoulas, Cecelia L. Crawford, Benjamin B. Kim, Peter N. Fedorka, Edward C. Mun, Sameer B. Murali, Kristi Reynolds, Robert E. Zane, Sami Alskaf

**Affiliations:** 1 Department of Research and Evaluation, Kaiser Permanente Southern California, Pasadena, California; 2 Departments of Health Services and Pharmacy, University of Washington, Seattle; 3 Kaiser Permanente Washington Health Research Institute, Seattle; 4 Comparative Health Outcomes, Policy, and Economics (CHOICE) Institute, Department of Pharmacy, University of Washington, Seattle; 5 Department of Surgery, University of Pittsburgh School of Medicine, Pittsburgh, Pennsylvania; 6 Regional Nursing Research Program, Kaiser Permanente Southern California, Pasadena; 7 Department of Surgery, Kaiser Permanente South Bay Medical Center, Harbor City, California; 8 Department of Surgery, Kaiser Permanente Ontario Medical Center, Ontario, Canada; 9 Department of Surgery, Kaiser Permanente South Bay Medical Center, Harbor City, California; 10 Center for Obesity Medicine & Metabolic Performance, Department of Surgery, University of Texas McGovern Medical School, Houston; 11 Division of Metabolic Obesity Medicine, Kaiser Permanente Panorama City Medical Center, Panorama City, California

## Abstract

**Importance:**

The comparative effectiveness of the most common operations in the long-term management of dyslipidemia is not clear.

**Objective:**

To compare 4-year outcomes associated with vertical sleeve gastrectomy (VSG) vs Roux-en-Y gastric bypass (RYGB) for remission and relapse of dyslipidemia.

**Design, Setting, and Participants:**

This retrospective comparative effectiveness study was conducted from January 1, 2009, to December 31, 2016, with follow-up until December 31, 2018. Participants included patients with dyslipidemia at the time of surgery who underwent VSG (4142 patients) or RYGB (2853 patients). Patients were part of a large integrated health care system in Southern California. Analysis was conducted from January 1, 2018, to December 31, 2021.

**Exposures:**

RYGB and VSG.

**Main Outcomes and Measures:**

Dyslipidemia remission and relapse were assessed in each year of follow-up for as long as 4 years after surgery.

**Results:**

A total of 8265 patients were included, with a mean (SD) age of 46 (11) years; 6591 (79.8%) were women, 3545 (42.9%) were Hispanic, 1468 (17.8%) were non-Hispanic Black, 2985 (36.1%) were non-Hispanic White, 267 (3.2%) were of other non-Hispanic race, and the mean (SD) body mass index (calculated as weight in kilograms divided by height in meters squared) was 44 (7) at the time of surgery. Dyslipidemia outcomes at 4 years were ascertained for 2168 patients (75.9%) undergoing RYGB and 3999 (73.9%) undergoing VSG. Remission was significantly higher for those who underwent RYGB (824 [38.0%]) compared with VSG (1120 [28.0%]) (difference in the probability of remission, 0.10; 95% CI, 0.01-0.19), with no differences in relapse (455 [21.0%] vs 960 [24.0%]). Without accounting for relapse, remission of dyslipidemia after 4 years was 58.9% (1279) for those who underwent RYGB and 51.9% (2079) for those who underwent VSG. Four-year differences between operations were most pronounced for patients 65 years or older (0.39; 95% CI, 0.27-0.51), those with cardiovascular disease (0.43; 95% CI, 0.24-0.62), or non-Hispanic Black patients (0.13; 95% CI, 0.01-0.25) and White patients (0.13; 95% CI, 0.03-0.22).

**Conclusions and Relevance:**

In this large, racially and ethnically diverse cohort of patients who underwent bariatric and metabolic surgery in clinical practices, RYGB was associated with higher rates of dyslipidemia remission after 4 years compared with VSG. However, almost one-quarter of all patients experienced relapse, suggesting that patients should be monitored closely throughout their postoperative course to maximize the benefits of these operations for treatment of dyslipidemia.

## Key Points

**Question:**

Do dyslipidemia outcomes up to 4 years after surgery differ between patients undergoing vertical sleeve gastrectomy (VSG) and Roux-en-Y gastric bypass (RYGB)?

**Findings:**

This comparative effectiveness study included 8265 racially and ethnically diverse patients who underwent VSG or RYGB and was conducted with a local instrumental variable approach to adjust for confounding in the choice of operation. Dyslipidemia remission was higher for patients who underwent RYGB (59%) than those who underwent VSG (52%) after 4 years, with high rates of relapse that were similar for RYGB and VSG during that time (21% vs 24%).

**Meaning:**

These findings suggest that patients should be monitored closely throughout their postoperative course to maximize the benefits of these operations for treatment of dyslipidemia.

## Teaser

This comparative effectiveness study evaluates outcomes associated with vertical sleeve gastrectomy vs Roux-en-Y gastric bypass for remission and relapse of dyslipidemia in a cohort of racially and ethnically diverse patients.

## Introduction

Obesity is tied to the etiology of cardiovascular disease (CVD) through its direct effect on glucose regulation and lipid metabolism. It is estimated that treating dyslipidemia alone (which includes any one of elevated low-density lipoproteins [LDL], triglycerides, total cholesterol [TC], and/or low high-density lipoproteins [HDL]) could lead to as much as a 30% reduction in mortality from CVD.^1^ Weight loss is often recommended as a treatment for dyslipidemia in adults with obesity^2^; however, weight loss and maintenance through lifestyle modification alone is challenging.^3^ In contrast, bariatric and metabolic surgeries result in sustained, long-term weight loss.^4,5^

Bariatric and metabolic surgeries also have promise as effective treatments to improve risk factors for CVD, such as diabetes^6,7^ and hypertension.^8,9^ The least studied of these risk factors is dyslipidemia. Most observational studies are short term (1-2 years after surgery) and have shown that dyslipidemia improved after surgery.^10,11^ The Longitudinal Assessment of Bariatric Surgery study found that Roux-en-Y gastric bypass (RYGB) was effective for improving dyslipidemia for 7 years after surgery.^12^ In addition to observational studies, 3 long-term randomized trials have been published, all of which found that after 5 years, bariatric and metabolic surgeries were superior to standard medical care with respect to triglyceride and HDL levels.^13,14,15^

There has been much less research on the comparative effectiveness of contemporary bariatric and metabolic operations for the treatment of dyslipidemia. One meta-analysis of randomized trials comparing RYGB and vertical sleeve gastrectomy (VSG), the most common operation in the US,^16^ found that remission of dyslipidemia was higher for patients who underwent RYGB vs VSG at 1 year (moderate certainty of evidence) and 5 years (low certainty of evidence).^11^ In another meta-analysis with both observational and randomized studies, RYGB was superior to VSG for improving dyslipidemia in the short term (1-2 years) with no differences in the longer term.^17^

The published literature on the comparative associations of bariatric and metabolic operations with dyslipidemia outcomes has several limitations: (1) there are few large, long-term (>2 years) comparative outcome studies in clinical settings where most patients would undergo surgery (rather than randomized trials) that reflect the current practice of primarily VSG operations; (2) to our knowledge, no examination exists of the rates of relapse of dyslipidemia after initial dyslipidemia remission; and (3) few studies have included populations with greater CVD risk that might benefit most from surgery, such as non-Hispanic Black patients. It is well documented that Black and Hispanic individuals are disproportionately affected by obesity and the resulting risk for CVD.^18,19^ Understanding the association between surgery and dyslipidemia outcomes in these racial and ethnic groups of patients would contribute substantially to reducing the burden of CVD in these populations. In addition, many studies do not incorporate information about how surgeons and patients make decisions regarding which operation to have, which can bias the comparative findings.^20^

To address these limitations, we compared the effectiveness of VSG and RYGB for dyslipidemia remission and relapse up to 4 years after surgery in the Effectiveness of Gastric Bypass vs Gastric Sleeve for Cardiovascular Disease (ENGAGE CVD) study cohort. The ENGAGE CVD study was designed to use rigorous comparative effectiveness methods and stakeholder engagement to understand decisions between RYGB and VSG so that we could account for these differences.^21^

## Methods

### Study Design

The ENGAGE CVD study design has been previously described in detail.^21^ Briefly, it was a retrospective comparative effectiveness study using a local instrumental variable approach to adjust for confounding in the choice of VSG or RYGB. The study period was January 1, 2009, to December 31, 2018. The International Society for Pharmacoeconomics and Outcomes Research (ISPOR) guidelines were used to report methods and outcomes.^22^

### Identification of the Study Population

Briefly, we used health plan data to identify adult members of a large integrated health care system with approximately 4.7 million members in the Southern California region of the US who underwent VSG or RYGB between January 1, 2009, and December 31, 2016 (N = 22 095). Follow-up ended on December 31, 2018. Eligibility for weight loss surgery within the health care system is based on national recommendations^23^ and includes only adults aged 18 years or older. Patients also had to complete a standardized preparation course offered by the health system in which they received their care 6 months before surgery. There was a recommendation for 5% to 10% total weight loss before surgery during this course.

During the study period, bariatric and metabolic operations were performed by 23 surgeons across 4 internal and 5 external practices. Long-term care was solely the responsibility of the health system while patients were members of the health plan. As part of the health system, all patients were monitored annually using a standardized protocol for weight loss or regain, health outcomes, and long-term complications. This study was approved by the targeted health system’s institutional review board for human participants. The study was determined to be exempt because of the low risk, and written informed consent from patients was not required.

**Figure 1.**
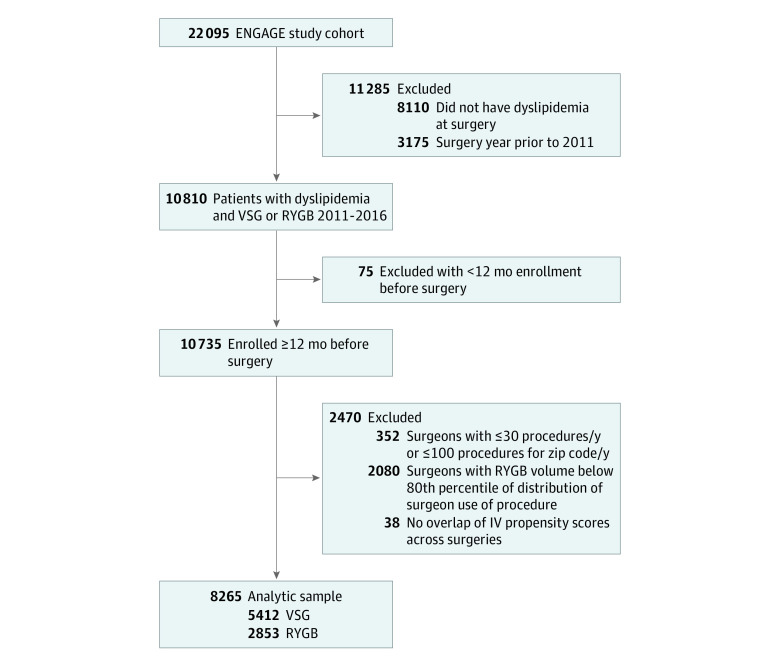
Flow Diagram for Selection of Patients With Dyslipidemia in the Effectiveness of Gastric Bypass vs Gastric Sleeve for Cardiovascular Disease (ENGAGE CVD) Cohort Study IV indicates instrumental variable; RYGB, Roux-en-Y gastric bypass; VSG, vertical sleeve gastrectomy.

Figure 1 shows the derivation of the analytic sample. Beginning with the larger cohort (N = 22 095), we restricted our analysis to patients with dyslipidemia at surgery (n = 13 985). Further exclusions were made for the instrumental variable (IV) analyses, which are detailed in eTable 1 in the Supplement. The final analytic sample included 8265 patients with dyslipidemia (5412 who underwent VSG and 2853 who underwent RYGB).

The definition of dyslipidemia at the time of surgery was created from previous research in this area^11,17,24,25^ as well as guidelines for outcomes research in bariatric and metabolic surgery from the American Society for Metabolic and Bariatric Surgery.^26^ Patients had to have at least 1 of the following at or before the date of surgery: LDL level of 160 mg/dL or greater, TC level of 240 mg/dL or greater, triglyceride levels of 200 mg/dL or greater, HDL level less than 40 mg/dL for men and less than 50 mg/dL for women, or any lipid-lowering medication used at the time of surgery (to convert LDL, TC, and HDL to millimoles per liter, multiply by 0.0259; to convert triglycerides to millimoles per liter, multiply by 0.0113). A patient was considered to be using lipid-lowering medication at the time of surgery if the last medication sold date before surgery plus 1.25 × days’ supply included the surgery date. This formula allowed for the inclusion of patients who did not take their medications exactly as instructed.^25^ The look-back period for lipid values was 5 years; however, we used the value closest to the date of surgery, which was typically within 12 months. A 5-year look-back period was used because the clinical practice guidelines of the organization stipulated lipids be measured every 5 years among adults with low CVD risk.

### Patient Demographic and Clinical Characteristics

Data for the study were obtained from patient electronic medical records and billing claims for outside services. Sex, age, and race and ethnicity were self-reported to clinical staff during routine care. Racial and ethnic categories included Hispanic, non-Hispanic Black, non-Hispanic White, and an “other” category that included American Indian or Alaska Native, Asian, Hawaiian or Pacific Islander, multiracial, other, and unrecorded. The Elixhauser Comorbidity Index^27^ was calculated using diagnosis codes. History of CVD was defined as having any of the following diagnoses in the 24 months before surgery: acute myocardial infarction, unstable angina, percutaneous coronary intervention, coronary artery bypass grafting, ischemic stroke, or hemorrhagic stroke. The *International Classification of Diseases*, *Ninth Revision *(*ICD-9*) and *International Statistical Classification of Diseases and Related Health Problems, Tenth Revision *(*ICD-10*) as well as Current Procedural Terminology codes used for these events are available on request.

### Outcome Measures

The definitions of dyslipidemia remission and relapse following surgery were consistent with previous research in this area^11,17,24,25^ and guidelines for outcomes research in bariatric and metabolic surgery created by the American Society for Metabolic and Bariatric Surgery^26^ and reflected the clinical practice guidelines in the health care setting between 2009 and 2016 to align with postoperative care at that time. Remission and relapse were determined in each year of follow-up. If a patient’s status did not change, it was carried forward to the next year.

Remission was determined starting 30 days after surgery. All the following criteria had to be met within each year of follow-up before a person was considered in remission: LDL level less than 160 mg/dL, TC level less than 240 mg/dL, triglyceride levels less than 200 mg/dL, HDL level of 40 mg/dL or greater for men or 50 mg/dL or greater for women, and no lipid-lowering medication used at the time of each laboratory value (using the medication sold date + [days’ supply × 1.25] formula to determine overlap with the laboratory value). The date at which all conditions were met was assigned as the date of remission.

Relapse in each year of follow-up was calculated only in patients who experienced remission. If a person started using a lipid-lowering medication, they would be considered relapsed for that year regardless of any laboratory values within that year. The date the medication was sold was used as the date of relapse. If there was no evidence of a lipid-lowering medication within that year of follow-up, a person would be considered relapsed if any of the following laboratory values were found: LDL level 160 mg/dL or greater, HDL level less than 40 mg/dL for men or less than 50 mg/dL for women, TC level 240 mg/dL or greater, or triglyceride levels 200 mg/dL or greater. The date of the first laboratory value that met these criteria was assigned as the date of relapse.

### Statistical Analysis

#### IV Approach

For the outcome analyses, we used a local IV (LIV) approach to balance measured and unmeasured confounders and covariates that could be reasons why a patient chose VSG or RYGB.^28,29,30,31,32,33^ To choose these confounders and covariates, we used previous literature in this area^4,5,6,7,8,34^ as well as extensive collaboration with an advisory board of bariatric and metabolic surgeons.^21^ Details about the LIV methods are provided in the eAppendix, eFigures 1-5, and eTables 2 and 3 in the Supplement. The confounders and covariates chosen from this process are shown in Table 1.

**Table 1.** Differences in Baseline Confounders and Covariates for Patients With Dyslipidemia Undergoing Vertical Sleeve Gastrectomy (VSG) and Roux-en-Y Gastric Bypass (RYGB) in the Effectiveness of Gastric Bypass vs Gastric Sleeve for Cardiovascular Disease Study Between Operation Type and Across Instrumental Variable (IV) Levels

Characteristic^a^	Mean (SD)		*P* value	Mean (SE)		*P* value^c^
	VSG (n = 5412)	RYGB (n = 2853)		<Median IV (n = 4055)^b^	≥Median IV (n = 4210)^b^	
No. of follow-up days after surgery	838 (807)	839 (812)	.30	NA	NA	NA
Age, y	45.8 (11.4)	47.0 (11.0)	<.001	46.0 (0.29)	46.4 (0.30)	.19
BMI	43.5 (6.5)	43.7 (6.9)	.12	43.5 (0.21)	43.7 (0.20)	.33
Elixhauser Comorbidity Score	0.94 (9.14)	2.35 (9.76)	<.001	1.30 (0.25)	1.56 (0.23)	.28
Cholesterol, mg/dL						
LDL	111.30 (35.34)	104.03 (36.05)	<.001	108.94 (0.90)	108.63 (0.88)	.73
HDL	43.56 (9.05)	43.04 (9.29)	.01	43.44 (0.25)	43.32 (0.22)	.60
Total	178.59 (39.53)	169.85 (41.96)	<.001	175.55 (0.67)	175.60 (.065)	.94
Triglycerides, mg/dL	154.36 (96.96)	163.39 (94.55)	<.001	155.29 (2.0)	159.82 (1.9)	.02
12 mo before surgery						
Weight change, lb	−17.6 (14.7)	−17.0 (14.1)	.06	−17.6 (0.27)	−17.1 (0.26)	.06
Scheduled visit attendance rate, %	76 (12)	77 (12)	.34	77 (0.70)	76 (0.68)	.15
No. of inpatient days	0.07 (0.39)	0.07 (0.32)	.87	0.07 (0.01)	0.08 (0.01)	.32
No. of emergency visits	0.35 (1.12)	0.36 (0.95)	.61	0.34 (0.02)	0.36 (0.02)	.38
Sex, No. (%)						
Women	4368 (80.7)	2223 (77.9)	.003	3236 (79.8)	3355 (79.7)	.98
Men	1044 (19.3)	630 (22.1)		819 (20.2)	855 (20.3)	
Hispanic, No. (%)	2267 (41.9)	1278 (44.8)	.01	1772 (43.7)	1760 (41.8)	.10
Non-Hispanic, No. (%)						
Black	1066 (19.7)	402 (14.1)	<.001	742 (18.3)	693 (17.1)	.17
White	1921 (35.5)	1064 (37.7)	.10	1456 (35.9)	1549 (36.8)	.09
Other	158 (2.9)	109 (3.8)	.004	85 (2.1)	181 (4.3)	<.001
Ever smoker, No. (%)	1855 (34.3)	1009 (35.4)	.32	1427 (35.2)	1431 (34.0)	.30
24 mo before surgery, No. (%)						
Gastroesophageal reflux disease	1641 (30.3)	1061 (37.2)	<.001	1289 (31.8)	1419 (33.7)	.09
Gastritis or duodenitis	904 (16.7)	147 (5.2)	<.001	624 (15.4)	413 (9.8)	<.001
Dyspepsia	211 (3.9)	130 (4.6)	.15	187 (4.6)	164 (3.9)	.06
Hiatal hernia	151 (2.8)	113 (4.0)	.004	142 (3.5)	122 (2.9)	.16
Sleep apnea	841 (15.5)	499 (17.5)	.02	661 (16.3)	678 (16.1)	.86
Type 2 diabetes	1530 (28.3)	1653 (57.9)	<.001	1525 (37.6)	1663 (39.5)	.10
Cardiovascular disease	207 (3.8)	126 (4.4)	.19	164 (3.9)	1773 (4.1)	.55
Never smoker	3557 (65.7)	1844 (64.6)	.32	2668 (65.8)	2732 (64.9)	.35
Chronic kidney disease	690 (12.7)	445 (15.6)	<.001	568 (14.0)	564 (13.4)	.49
Severe mental illness	291 (5.4)	182 (6.4)	.06	231 (5.7)	244 (5.8)	.92
Severe depression or anxiety	341 (6.3)	209 (7.3)	.08	264 (6.5)	286 (6.8)	.59
Mild to moderate depression or anxiety	2310 (42.7)	1201 (42.1)	.61	1695 (41.8)	1819 (43.2)	.21
BMI ≥50	789 (14.6)	465 (16.3)	.04	612 (15.1)	644 (15.3)	.82
Aspirin use before surgery, No. (%)						
12 mo	978 (18.1)	889 (31.2)	<.001	916 (22.6)	951 (22.6)	.97
3 mo	645 (11.9)	583 (20.4)	<.001	661 (16.3)	560 (13.3)	<.001
NSAID use before surgery, No. (%)						
12 mo	2387 (44.1)	1250 (43.8)	.80	1768 (43.6)	1869 (44.4)	.49
3 mo	827 (15.3)	411 (14.4)	.89	620 (15.3)	619 (14.7)	.46

Abbreviations: BMI, body mass index (calculated as weight in kilograms divided by height in meters squared); HDL, high-density lipoproteins; LDL, low-density lipoproteins; NA, not applicable; NSAID, nonsteroidal anti-inflammatory drug.

SI conversion factors: To convert HDL, LDL, and TC to millimoles per liter, multiply by 0.0259; to convert triglycerides to millimoles per liter, multiply by 0.0113.

^a^ All values were recorded at the time of surgery unless otherwise noted.

^b^ The IV was the rate of RYGB in the previous year for each surgeon.

^c^ After controlling for year of surgery and 3-digit zip code indicators, zip code–level surgery volume, and surgeon-specific caseload in previous year. If the *P* value for the IV median comparison was not significant, the variable was balanced between VSG and RYGB.

The outcome was modeled as a 3-level ordinal variable (original [did not experience remission], remission, or relapse) with 2 separate logit models using generalized estimating equations with a logit link function and exchangeable correlation structure.^35,36^ The eAppendix in the Supplement provides detailed descriptions of these models. Comparative results are presented as the average difference in adjusted probabilities between operations (RYGB – VSG) with 95% CIs. A positive difference favored RYGB. To compare our results with those reported in the literature, we combined the rates of remission and relapse into a descriptive statistic referred to as remission without accounting for relapse. Statistical significance was determined by 95% CIs not overlapping 0 and *P* values <.05. *P* values were calculated as part of the generalized estimating equations.

#### Heterogeneity of Treatment Effects

The overall results were then aggregated to form the population mean, and subgroup-specific mean treatment effects^29,37,38,39^ were used to examine the differences between RYGB and VSG for dyslipidemia remission and relapse across age (<65 years or ≥65 years), body mass index (BMI; calculated as weight in kilograms divided by height in meters squared; categories were <50 or ≥50), having a history of CVD (yes or no), being a smoker (ever smoked or never smoked), and race and ethnicity (Hispanic, non-Hispanic Black, or non-Hispanic White; other racial and ethnic groups were not included in these analyses because the sample was very small and heterogeneous, making any findings difficult to interpret). All standard errors were calculated using nonparametric bootstrapping and allowed for clustering of individual outcomes over time.

### Sensitivity Analyses

Lipid levels were measured at different intervals for different patients. There was a mean of 250 days between measures. Because it was possible to complete single laboratory orders (eg, a single triglyceride test instead of a lipid panel that included a triglyceride test), not all lipid results were available at the same time with the same frequency during follow-up. To address this issue, we linearly interpolated lipid levels at regular intervals if patients had actual measured values before and after the interpolated time frame. Originally, our findings included a 5-year point estimate for dyslipidemia outcomes. However, the end of the follow-up period (December 31, 2018) resulted in few patients with actual measured values after 5 years. Thus, our main findings were limited to a 4-year point estimate (most patients had measures after 4 years). We present 5-year findings throughout the Supplement for descriptive purposes only.

To compare our findings with those of other literature, we performed 3 sensitivity analyses: (1) a standard multivariate regression, (2) a standard inverse probability weighted propensity score, and (3) a standard IV approach using a 2-stage residual inclusion approach (eFigures 7 and 8 in the Supplement).^40,41^ We also compared the dyslipidemia remission and relapse results we obtained with the LIV sample (8265 patients) with the larger sample (10 383) using propensity scores before applying the LIV exclusions (eFigure 9 in the Supplement). All analyses were conducted from January 1, 2018, to December 31, 2021, in Stata, version 15.1 (StataCorp LLC).

## Results

### Study Population

Table 1 presents descriptive statistics for the sample used for the analyses (2853 patients who underwent RYGB and 5412 who underwent VSG) before and after adjustment for the IV. Patients had a mean (SD) age of 46 (11) years, 6591 (79.8%) were women and 1674 (20.2%) were men, 3545 (42.9%) were Hispanic, 1468 (17.8%) were non-Hispanic Black, 2985 (36.1%) were non-Hispanic White, 267 (3.2%) were of other non-Hispanic race, and the mean (SD) BMI was 44 (7) at the time of surgery. The IV was significantly predictive of having a RYGB operation (F statistic, 373; *P* < .001). When confounders and covariates were stratified by the IV median, the balance improved for most covariates (Table 1). All variables from Table 1 were adjusted for in the outcome analyses.

### Follow-up

The overall 4-year retention rate was 74.6% (6167 patients), including 2168 (75.9%) who underwent RYGB and 3999 (73.9%) who underwent VSG. A total of 2098 patients were lost to follow-up or censored because of lost membership in the health plan (1594 [75.9%]), death (63 [3.0%]), and not having lipid measures at any time during follow-up (441 [21.0%]). There were no differences between bariatric and metabolic operations in loss to follow-up (eFigure 10 in the Supplement).

### Dyslipidemia Remission and Relapse

**Figure 2.**
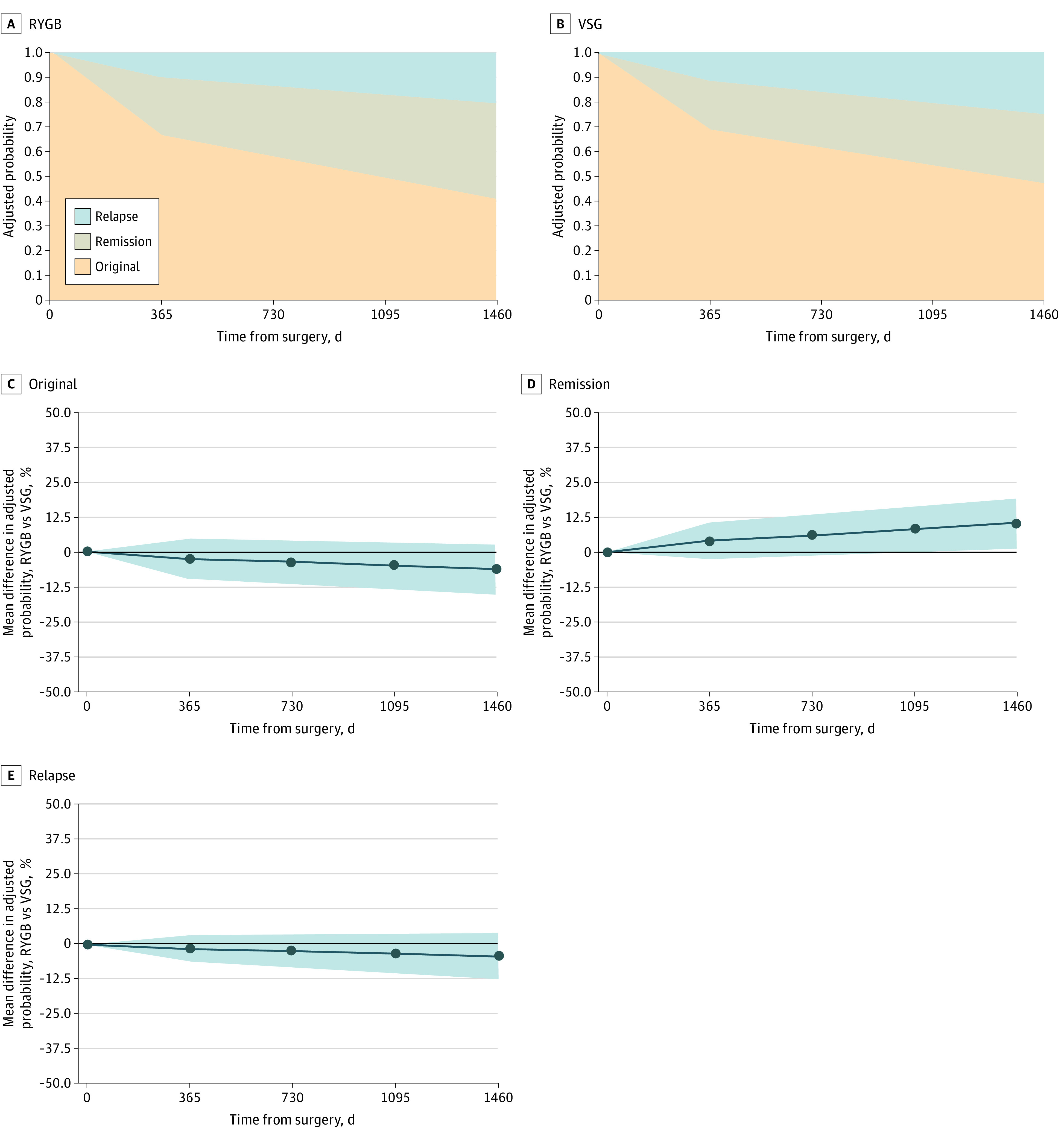
Dyslipidemia Status for Patients Following Vertical Sleeve Gastrectomy (VSG) or Roux-en-Y Gastric Bypass (RYGB) in the Effectiveness of Gastric Bypass vs Gastric Sleeve for Cardiovascular Disease Cohort Study An original status refers to patients who continued to have dyslipidemia throughout the follow-up period and never experienced remission (or relapse). Data are presented as adjusted probabilities in panels A and B and the average difference in adjusted probabilities between RYGB and VSG with 95% CIs (blue shading) in panels C, D, and E. 95% CIs that did not overlap 0 were considered statistically significant.

Dyslipidemia outcomes at 4 years were ascertained for 2168 patients (75.9%) undergoing RYGB and 3999 (73.9%) undergoing VSG and are shown in Table 2 and Figure 2A and B. Remission rates were significantly different between operations at 4 years (38.0% [824 patients] among those who underwent RYGB and 28.0% [1120 patients] among those who underwent VSG; difference in the probability of remission, 0.10; 95% CI, 0.01-0.19). There were no significant differences in relapse rates between RYGB and VSG throughout follow-up (21.0% [455 patients] among those who underwent RYGB and 24.0% [960 patients] among those who underwent VSG at 4 years). Without accounting for relapse, at 4 years, 1279 patients (58.9%) who underwent RYGB and 2079 patients (51.9%) who underwent VSG experienced dyslipidemia remission.

**Table 2.** Dyslipidemia Status for Patients After Having RYGB or VSG in the Effectiveness of Gastric Bypass vs Gastric Sleeve for Cardiovascular Disease Cohort Study^a^

Days	Adjusted probabilities								Average difference in adjusted probabilities, RYGB − VSG (95% CI)		
	RYGB				VSG						
	No. of patients	Original	Remission	Relapse	No. of patients	Original	Remission	Relapse	Original	Remission	Relapse
365	2848	0.67	0.23	0.10	5412	0.69	0.20	0.11	−0.02 (−0.09 to 0.05)	0.03 (−0.03 to 0.11)	−0.01 (−0.06 to 0.03)
730	2831	0.59	0.28	0.13	5366	0.62	0.23	0.15	−0.03 (−0.12 to 0.04)	0.05 (−0.01 to 0.14)	−0.02 (−0.08 to 0.03)
1095	2481	0.50	0.33	0.17	4697	0.54	0.26	0.20	−0.05 (−0.14 to 0.04)	0.07 (−0.00 to 0.17)	−0.03 (−0.10 to 0.04)
1460	2168	0.41	0.38	0.21	3999	0.48	0.28	0.24	−0.07 (−0.15 to 0.03)	0.10 (0.01 to 0.19)	−0.03 (−0.13 to 0.04)

Abbreviations: RYGB, Roux-en-Y gastric bypass; VSG, vertical sleeve gastrectomy.

^a^ Original refers to patients who continued to have dyslipidemia throughout the follow-up period and never experienced remission (or relapse). To obtain the rate of remission without accounting for relapse, the remission and relapse rates should be combined (ie, at 1825 days [4 years] 59% for RYGB and 52% for VSG). 95% CIs not overlapping 0 are considered statistically significant.

### Lipid Levels

**Figure 3.**
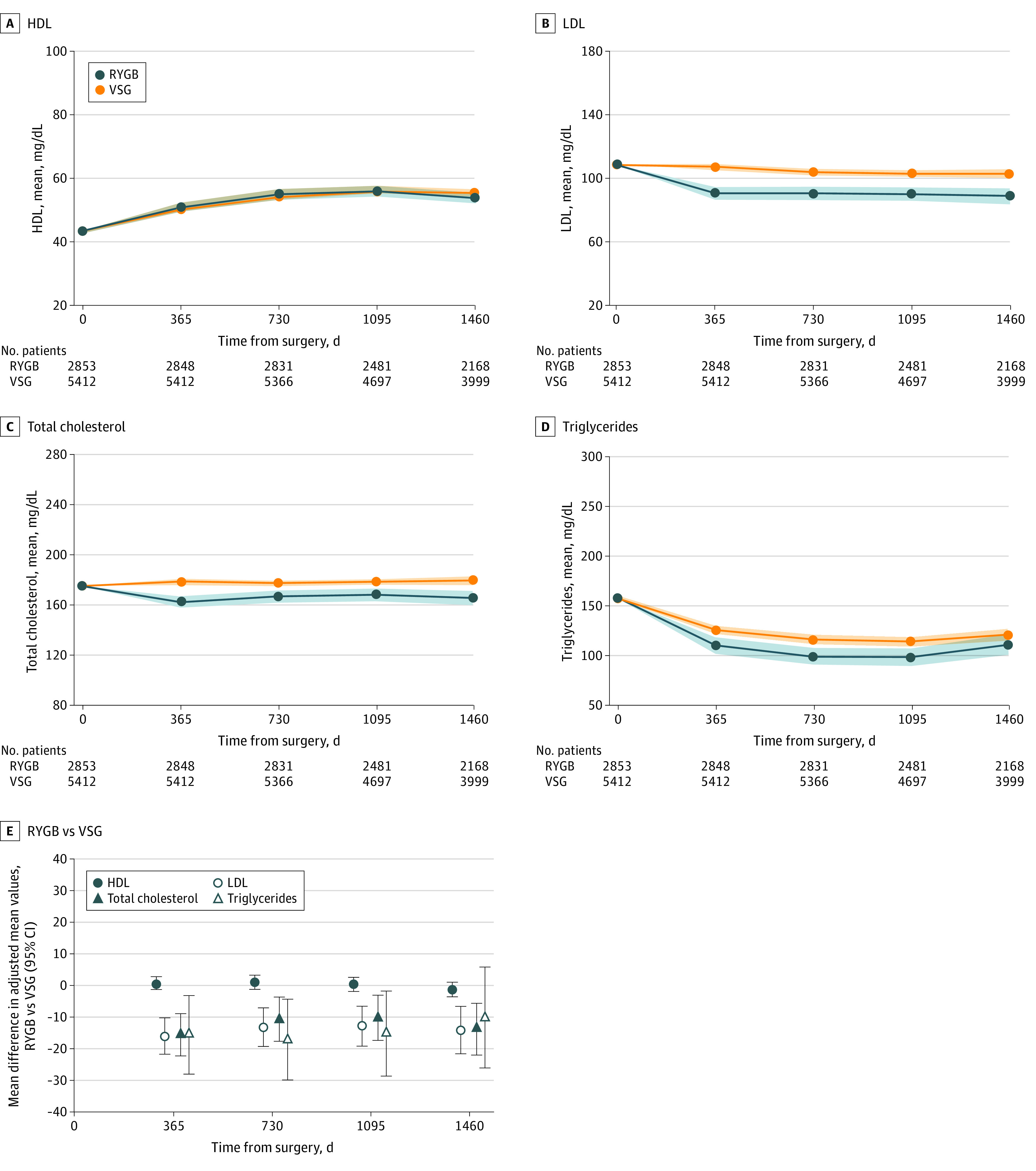
Lipid Levels Between Vertical Patients Undergoing Sleeve Gastrectomy (VSG) and Roux-en-Y Gastric Bypass (RYGB) in the Effectiveness of Gastric Bypass vs Gastric Sleeve for Cardiovascular Disease Cohort Study Data are presented at baseline and in each year of follow-up for unadjusted mean levels (panels A-D) and the average difference in adjusted mean values between RYGB and VSG with 95% CIs (panel E). 95% CIs that did not overlap 0 were considered statistically significant. To convert high-density lipoprotein (HDL), low-density lipoprotein (LDL), and total cholesterol to millimoles per liter, multiply by 0.0259; to convert triglycerides to millimoles per liter, multiply by 0.0113.

Findings for unadjusted mean levels of LDL, HDL, triglycerides, and TC in each year of follow-up by operation are shown in Figures 3A-3D and eTable 4 in the Supplement. In general, HDL levels increased, and LDL and triglyceride levels decreased over time. The adjusted average mean differences between RYGB and SG are shown in Figure 3D and eTable 4 in the Supplement. At all years of follow-up, there was a significantly greater difference in mean levels of LDL and TC favoring RYGB. These differences were significant only for triglyceride levels in years 1 to 3 and were not significant at any time for HDL levels.

### Heterogeneity of Treatment Effects

For years 1 to 4 of follow-up, patients aged 65 or older who underwent RYGB had a significantly higher probability of dyslipidemia remission and a significantly lower probability of relapse when compared with patients aged 65 or older who underwent VSG (the difference in the probability of remission at 4 years was 0.39; 95% CI, 0.27-0.51; and the difference in the probability of relapse at 4 years was −0.23; 95% CI, −0.37 to −0.08) (eFigure 11A in the Supplement). These findings were similar for patients with CVD at the time of surgery (the difference in the probability of remission at 4 years was 0.43; 95% CI, 0.24-0.62; and the difference in the probability of relapse at 4 years was −0.20; 95% CI, −0.39-0.00) (eFigure 11C in the Supplement). Finally, for non-Hispanic Black patients, there was a significantly greater probability of dyslipidemia remission with RYGB in years 3 and 4 when compared with VSG (the difference in the probability of remission at 4 years was 0.13; 95% CI, 0.01-0.25) (eFigure 11E in the Supplement), whereas non-Hispanic White patients experienced this significant difference in dyslipidemia remission in years 2 to 4 of follow-up (the difference in the probability of remission at 4 years was 0.13; 95% CI, 0.03-0.22) (eFigure 11E in the Supplement). Other comparisons were not statistically significant.

### Sensitivity Analyses

Using interpolated and smoothed mean lipid values instead of raw values did not change any of the findings (eFigure 6 in the Supplement). In addition, results were similar when LIV methods were compared with other approaches that addressed only observed confounding (eg, regression or propensity score approaches [eFigures 7 and 8 in the Supplement]). Finally, the findings were similar when we compared results from propensity score weighting in the LIV sample (8265 patients) with the larger sample (10 383 patients) (eFigure 9 in the Supplement).

## Discussion

In one of the largest racially and ethnically diverse samples (63% non-Hispanic Black or Hispanic) of patients undergoing the 2 most common weight loss operations performed today in clinical practice, we found that dyslipidemia remission, without accounting for relapse, was 59% for RYGB compared with 52% for VSG after 4 years of follow-up. These differences were most pronounced for patients aged 65 years or older, those with CVD at the time of surgery, and non-Hispanic White patients. For non-Hispanic Black patients, these differences were seen only in years 3 and 4 of follow-up. Almost one-quarter of patients regardless of operation experienced a relapse of their dyslipidemia (21% for RYGB and 24% for VSG) during this same period of follow-up, resulting in lower rates of durable dyslipidemia remission (38% for RYGB and 28% for VSG). We also found that patients who underwent RYGB had significantly lower TC and LDL levels than patients who underwent VSG at every year of follow-up. Differences between patients who underwent RYGB or VSG were only seen in years 1 to 3 for triglyceride levels, and there were no differences for HDL levels.

Our findings are somewhat supported by previous research comparing VSG and RYGB. In the only meta-analysis that included observational studies,^17^ improvement or remission of dyslipidemia was more likely with RYGB than VSG for as long as 3 years after surgery, but there were no significant differences after 3 years. Our study is one of the first to show a high rate of dyslipidemia relapse among all patients undergoing either RYGB or VSG (21% for RYGB and 24% for VSG). This finding has important implications for future work in this area, highlighting the need to focus on durable CVD risk factor remission.

Despite these relapse rates, there may still be health benefits for patients from any period of remission. Although not tested in the current study, previous work has shown that even if patients experienced a relapse of their diabetes after surgery, they still had a lower risk of microvascular complications from diabetes than patients who never experienced remission.^42^ It is possible that even if patients experience relapse of their dyslipidemia, any period of remission could also confer long-term benefits for CVD mortality. This possibility remains to be tested.

It is also important to consider complication rates for VSG and RYGB when making recommendations about a particular operation for any one patient. In previous reports of the safety outcomes for patients in the ENGAGE CVD cohort,^43,44^ RYGB had higher rates of complications over a 5-year period when compared with VSG. These complications should be considered in the decision-making process between bariatric and metabolic operations for severe obesity and CVD risk factor remission.

### Limitations and Strengths

Our study had several limitations, most notably that it was a retrospective observational design with nonrandom assignment to operation. However, we used the LIV approach to address confounding and account for the surgeons’ and patients’ decisions between bariatric and metabolic operations.^21^ Another limitation, specific to the use of the LIV approach, was that patients were excluded for this analysis. The eAppendix in the Supplement includes a full discussion of these methods and why these patients were excluded. However, when we used more traditional propensity score–based analyses that included these participants, the results were very similar (eFigures 7 and 8 in the Supplement).

In addition, we also did not test whether changes in body weight (loss and regain) were responsible for dyslipidemia remission and relapse. Unfortunately, mediation analysis in this setting is fraught with bias.^45^ We have applied advanced methods to deal with the endogeneity of surgery assignments because this was the a priori purpose of the study. To provide appropriate recommendations about the role of surgical weight loss and comorbidity resolution on dyslipidemia outcomes, we would need to design a separate study to address the question of what factors mediated our findings. Future research should explore the mechanisms responsible for changes in dyslipidemia remission and relapse following bariatric and metabolic surgery.

Despite these limitations, our study has notable strengths. To our knowledge, the ENGAGE CVD study is one of the largest and most diverse (61% of the cohort is non-Hispanic Black or Hispanic) observational cohort studies in a large clinical setting on the comparative effectiveness of VSG and RYGB for dyslipidemia remission and relapse with 4 years of follow-up. We used rigorous IV methods to overcome selection bias in choice of operation. Our findings were consistent in sensitivity analyses using different methods and were supported by the limited observational studies in this area.^12,17^

## Conclusions

In this comparative effectiveness study, we found that RYGB was associated with higher rates of dyslipidemia remission than VSG after 4 years of follow-up. Nevertheless, 21% of patients who underwent RYGB and 28% of patients who underwent VSG experienced a relapse of their dyslipidemia in the 4-year follow-up period, indicating that bariatric and metabolic surgery alone does not guarantee a patient will maintain remission. Future studies are warranted to understand why patients experience dyslipidemia relapse after weight loss surgery.

## Notes

Supplement. **eAppendix.** Additional statistical methods **eTable 1.** Detailed inclusions and exclusions for the Effectiveness of Gastric Bypass vs. Gastric Sleeve for Cardiovascular Disease (ENGAGE CVD) study **eFigure 1.** Distribution of instrumental variable (IV) and the balance of normalized observed confounders over IV levels **eTable 2.** Differences in patient characteristics for central 10 – 90 versus peripheral 20 percentile of surgeon-specific rates **eFigure 2.** Balance of covariate z-scores (a) across treatment, and (b) across IV levels **eFigure 3.** Standardized mean difference for each covariate across treatment versus across median of the IV (i.e. below and above the median) **eTable 3.** Factors affecting RYGB selection (first-stage IV regression) **eFigure 4.** Distribution of IV-propensity score by treatment **eFigure 5.** Standardized mean difference for each covariate across treatment with and without propensity score-based weighting **eFigure 6.** Trends in lipid levels with interpolation and raw data without interpolation **eTable 4.** Lipid levels for vertical sleeve gastrectomy (VSG) and Roux-en-Y Gastric Bypass (RYGB) **eFigure 7.** Average effects on dyslipidemia status by different analytic techniques **eFigure 8.** Average effects on lipid levels by different analytic techniques **eFigure 9.** Comparing propensity-score-based analysis results on final sample used in LIV analysis versus on full sample with no trimming due to LIV analysis **eFigure 10.** Hazard of attrition by bariatric surgery operation due to (a) disenrollment or (b) death **eFigure 11.** Adjusted proportions of patients with original dyslipidemia, remission, or relapse over 5-years for the two bariatric surgical operations by subgroups
eReferences
