# Supplementary material for: Remission and Relapse of Dyslipidemia After Vertical Sleeve Gastrectomy vs Roux-en-Y Gastric Bypass in a Racially and Ethnically Diverse Population
Source: JAMA Netw Open. 2022 Sep 28;5(9):e2233843. doi: 10.1001/jamanetworkopen.2022.33843 (PMC9520365; doi:10.1001/jamanetworkopen.2022.33843)
Supplement: Supplement. — eAppendix. Additional statistical methods eTable 1. Detailed inclusions and exclusions for the Effectiveness of Gastric Bypass vs. Gastric Sleeve for Cardiovascular Disease (ENGAGE CVD) study eFigure 1. Distribution of instrumental variable (IV) and the balance of normalized observed confounders over IV levels eTable 2. Differences in patient characteristics for central 10 – 90 versus peripheral 20 percentile of surgeon-specific rates eFigure 2. Balance of covariate z-scores (a) across treatment, and (b) across IV levels eFigure 3. Standardized mean difference for each covariate across treatment versus across median of the IV (i.e. below and above the median) eTable 3. Factors affecting RYGB selection (first-stage IV regression) eFigure 4. Distribution of IV-propensity score by treatment eFigure 5. Standardized mean difference for each covariate across treatment with and without propensity score-based weighting eFigure 6. Trends in lipid levels with interpolation and raw data without interpolation eTable 4. Lipid levels for vertical sleeve gastrectomy (VSG) and Roux-en-Y Gastric Bypass (RYGB) eFigure 7. Average effects on dyslipidemia status by different analytic techniques eFigure 8. Average effects on lipid levels by different analytic techniques eFigure 9. Comparing propensity-score-based analysis results on final sample used in LIV analysis versus on full sample with no trimming due to LIV analysis eFigure 10. Hazard of attrition by bariatric surgery operation due to (a) disenrollment or (b) death eFigure 11. Adjusted proportions of patients with original dyslipidemia, remission, or relapse over 5-years for the two bariatric surgical operations by subgroups eReferences [file jamanetwopen-e2233843-s001.pdf]

Supplemental Online Content

Coleman KJ, Basu A, Barton LJ, et al. Remission and relapse of dyslipidemia after vertical sleeve gastrectomy vs Roux-en-Y gastric bypass in a racially and ethnically diverse population. *JAMA Netw Open*. 2022;5(9):e2233843. doi:10.1001/jamanetworkopen.2022.33843

eAppendix. Additional statistical methods
eTable 1. Detailed inclusions and exclusions for the Effectiveness of Gastric Bypass vs. Gastric Sleeve for Cardiovascular Disease (ENGAGE CVD) study
eFigure 1. Distribution of instrumental variable (IV) and the balance of normalized observed confounders over IV levels
eTable 2. Differences in patient characteristics for central 10 – 90 versus peripheral 20 percentile of surgeon-specific rates
eFigure 2. Balance of covariate z-scores (a) across treatment, and (b) across IV levels
eFigure 3. Standardized mean difference for each covariate across treatment versus across median of the IV (i.e. below and above the median)
eTable 3. Factors affecting RYGB selection (first-stage IV regression)
eFigure 4. Distribution of IV-propensity score by treatment
eFigure 5. Standardized mean difference for each covariate across treatment with and without propensity score-based weighting
eFigure 6. Trends in lipid levels with interpolation and raw data without interpolation
eTable 4. Lipid levels for vertical sleeve gastrectomy (VSG) and Roux-en-Y Gastric Bypass (RYGB)
eFigure 7. Average effects on dyslipidemia status by different analytic techniques
eFigure 8. Average effects on lipid levels by different analytic techniques
eFigure 9. Comparing propensity-score-based analysis results on final sample used in LIV analysis versus on full sample with no trimming due to LIV analysis
eFigure 10. Hazard of attrition by bariatric surgery operation due to (a) disenrollment or (b) death
eFigure 11. Adjusted proportions of patients with original dyslipidemia, remission, or relapse over 5-years for the two bariatric surgical operations by subgroups
eReferences

This supplemental material has been provided by the authors to give readers additional information about their work.

© 2022 Coleman KJ et al. *JAMA Network Open.*

eAppendix. Additional statistical methods

Analytical Data and Detailed Exclusions

The ENGAGE cohort consisted of 22,095 patients at a large integrated health system in Southern California, who underwent sleeve gastrectomy (SG) or Roux-en-Y gastric bypass (RYGB) from 2009-2016. We included patients with dyslipidemia at surgery (n = 13,985). We restricted the sample to patients receiving surgery after 2010 (n = 10,810), when patients had the option to receive either RYBG or SG. At least one year of follow-up was required (n = 10,735).

For the IV analysis, we excluded patients with their physicians having low caseload counts based on which the IV was calculated (n = 352). The final exclusions come from exploration of instrumental variables (IV) and the appropriate segment of the real-world data where a pseudo experiment can be exploited through the use of IVs (**eTable 1**). Specifically, how we arrived at these numbers is described below.

Analytical Methods

Our primary analysis used a local IV approach to estimate person-centered treatment effects (PeT), along with other average treatment effects. Instrumental variables mimic a pseudo-randomization approach to establishing causal effects in healthcare observational research[1] and can address both observed and unobserved confounding. Unlike traditional IV approaches that estimate an effect on the marginal patient induced to select a different treatment due to the instrument, local IV approaches use a continuous IV to estimate the effect on every margin in the patient population.[2] In this way, not only can one address confounding by indication but also the treatment effect heterogeneity can be examined. We report PeT effects (details below),[3,4,5] which are individualized treatment effects for each person in our sample and can be easily aggregated to study population average treatment effects and also sub-group-specific average effects. A clinically intuitive description of these methods has been recently published.[5]

In addition, we conducted standard IV analysis using a two-stage residual inclusion approach and the standard inverse-probability weighted propensity score analysis as sensitivity

© 2022 Coleman KJ et al. *JAMA Network Open.*

analyses to provide comparability to the work that has been done comparing the effectiveness of SG vs RYGB for health outcomes. We now describe these methods in detail.

Local Instrumental Variable Method and Person-Centered Treatment (PeT) Effects

***Instrumental variable selection and testing*.** The LIV methods are designed to mimic the conditions of random assignment of treatments occurring in real-world clinical settings. An IV must be correlated with the exposure (choice of bariatric operation) but not associated with the outcome (dyslipidemia remission/relapse) except through its correlation with the exposure. The IV chosen for the current study was based on the stakeholder engagement process mentioned earlier,[19] and was the rate of use of RYGB by each physician during the 12 months prior to each patient's bariatric surgery (RYGB PP) after conditioning on 3-digit zip code and year of surgery. The use of this IV implies that patients within the same 3-digit zip code in the same chronological year were (pseudo) randomized to receive different operations based on having different surgeons (surgeons traditionally do not practice outside of a service region because of the need for access to assigned operating room time) with different preferences between RYGB vs SG. If such pseudo-randomization is occurring in practice, then the IV should be independent of all confounders, although we only can test this assumption using the observed covariates.

We observed a substantial reduction of observed covariate imbalance across most levels of the RYGB PP IV, especially for the central 80th percentile of the distribution (**eFigure 1**). For this Figure, normalization based was on computing a z-score for each covariate by demeaning it and dividing by its standard deviation. A lowess smoother was used to examine the levels of z-score across IV. This gives us confidence that such an approach will also help reduce imbalances in unobserved confounders, especially among those patients who are going to see surgeons in the central 80th percentile of the RYGB PP IV distribution.

© 2022 Coleman KJ et al. *JAMA Network Open.*

We found that compared to patients who were treated by surgeons within the central 80th percentile of RYGB PP, patients treated by surgeons outside the central 80th percentile of RYGB PP had slightly higher weight loss before surgery, were more likely to be non-Hispanic white, less likely to have sleep apnea, or severe anxiety, and more likely to have gastric duodenitis, chronic kidney disease (CKD) or gastroesophageal reflux disease (GERD) (**eTable 2**).

To maintain the highest validity of the RYGB PP IV, we restricted our analytical sample to patients treated by surgeons in the central 80th percentile (exclude N= 2,080). Similar methods to select a valid IV that best mimicked the pseudo-randomization of treatments have been used in the literature.[6,7,8,9] An additional 38 patients were excluded because they did not have a match receiving the opposite treatment but with the same IV-based propensity score to receive RYGB. The final sample consisted of 8,265 patients who had SG (N= 5,412) or RYGB (N = 2,853). At the time of surgery, compared to SG, RYGB patients were more likely to be male or Hispanic, had a higher Elixhauser comorbidity score, higher rates of GERD, T2DM, chronic kidney disease, and lower rates of gastric duodenitis.

Improvement in the balance of all the risk factors over IV levels compared to across treatment in our final sample are illustrated in **eFigures 2a-b** and **eFigure 3**. In **eFigure 2a-b**, each covariate was converted to a z score similar to what was used in **eFigure 1**; **eFigure 2a** shows the imbalance of these z scores across treatment, while **eFigure 2b** shows the imbalance of the same z-scores across IV levels, both conditioned on total volume of prescriptions, year, and 3-digit zip code fixed effects. The balance across IV was improved for all covariates.

We also looked at how the standardized mean difference for each covariate across treatment groups compared to that across the median of the IV. **eFigure 3** illustrates this reduction of the standardized mean difference attained by the IV variable.

These explorations of balance improvement in baseline risk factors across the IV variable (without any additional adjustments) suggest that the IV may be acting as a pseudo-randomization mechanism that not only reduces imbalance in observed risk factors without any

adjustment, but might also reduce imbalance in unobserved risk factors, thereby increasing the accuracy with which we can estimate a causal treatment effect.

Person-centered treatment effects for SG vs RYGB. To understand our choice of the local IV estimator (which we describe below), it is important to understand the context of selection bias or confounding by indication in the presence of treatment effect heterogeneity. There are three key concepts to understand in this situation: 1) "essential heterogeneity", 2) marginal treatment effects, and 3) person-centered treatment effects.

"Essential heterogeneity": Essential heterogeneity is a phenomenon that demands the used of methods such as local instrumental variable approaches to produce estimates for interpretable treatment effect parameters. It is often difficult to discount the idea, unless evidence shows otherwise, that there is substantial variability in the case-mix of patients receiving RYGB versus SG, and the incremental effectiveness of RYGB over SG may be heterogeneous. A further challenge is that the selection of patients for a particular modality of surgery may be driven according to risk factors that modify the effectiveness of RYGB over SG and vice-versa. Many of these factors, such as the patients' pre-admission health status may remain unmeasured in the data at hand. Together, when treatment effects of RYGB over SG are heterogeneous over unobserved confounders, the phenomenon is termed 'essential heterogeneity'. In this situation, most traditional methods for comparative effectiveness have limitations. Methods that rely on selection on observables (regression methods, propensity score-based methods) do not address biases due to unobserved confounders.

Traditional IV methods, that aim to address both observed and unobserved confounders, estimate a local average treatment effect parameter that is often not interpretable, or of clinical relevance. In particular, the resultant estimate only applies to the ill-defined subgroup who would have switched treatment modality according to a change in the level of the instrument, i.e. compliers.[10,11,12,13,14,15] Who the compliers are with respect to physician preference for a surgery is difficult to determine, and whether the effect of RYGB over SG for these patients would apply to other patients is difficult to say.

© 2022 Coleman KJ et al. *JAMA Network Open.*

We address these concerns with a recently developed econometric methodology of local instrumental variable (LIV) method that uses an (IV to address selection biases in observational studies and establish marginal treatment effects that can be aggregated to form PeT effects. PeT effects represent an average treatment effect for each person in the data, conditioning on their levels of risk factors, and accounting for their individualized distribution of unobserved heterogeneity. Consequently, such individualized effects can help study a variety of distributional questions on effectiveness, such as examining the benefits and harms of RYGB versus SG and identifying subgroups that are most likely to benefit from such care.

"Marginal treatment effects (MTEs)": MTEs are the treatment effects for those individuals for whom the influence of the observed characteristics (say, baseline BMI and the surgery preference of the physician), balance with the influence of the unobserved confounders (medical history) on the decision to use RYGB, such that the physician is indifferent between RYGB and SG. To estimate an MTE, LIV methods compare the outcomes of two groups of, similar patients (say BMI 40 kg/m^2), where one group sees a surgeon whose historical rate of RYGB preference is d and the other groups sees another surgeon with preference d+ε, with ε representing a slight increase in the preference for RYGB. These two groups of patients should be identical with respect to the distribution of their risk factors (observed and unobserved) provided physician preference is independent of all risk factors affecting outcomes. This independence assumption will hold if physician preference is a valid IV. Therefore, any difference in average outcomes between these two groups is only driven by the difference in the receipt of RYGB or SG, for this margin of patients where the clinicians were indifferent between RYGB and SG but were nudged to select RYGB by the small perturbation of the IV, i.e., physician preference.

For this margin of patients, we can quantify a normalized level of unobserved confounders that was sufficient to balance their observed confounders at the considered level of physician preference (d).[2,3] Here, normalized means a scalar score that represents a balancing score for unobserved risk factors, irrespective of their empirical distributions. One can think of the normalized level of unobserved confounders as the propensity to use SG based on unobserved confounders. For marginal patients, the propensity to use RYGB equals the propensity to use

© 2022 Coleman KJ et al. *JAMA Network Open.*

SG. The difference in average outcomes between the two groups of similar patients (e.g. BMI 40 kg/m^2) represents the MTE for those patients at that particular normalized level of unobserved confounders.

Similarly, for another dyad of physician preferences, d' and d'+ ε, one can estimate another MTE at another normalized level of unobserved confounder. In this way, a full schedule of MTEs can be estimated that vary over the unobserved confounder levels (e.g., medical history) given the level of the observed confounders (e.g., BMI), as long the physician preference (e.g., the IV) can take on a large number of distinct values; it is continuous. MTEs can be calculated by considering different values of the observed covariates, which will imply different values of the normalized unobserved confounders.

"Person-centered treatment (PeT) effects": The MTEs can be aggregated to study heterogeneity in effects using PeT effects. PeT effects are obtained by averaging the MTEs over only the normalized levels of the unobserved confounder (i.e., medical history) that conforms with the observed decision whether or not to use RYGB. Intuitively, if based on a patient's observed information (i.e., BMI) it is unlikely that they would receive RYGB, but we observe that they in fact do receive RYGB, this conveys useful information about their unobserved confounders (i.e., medical history). Accordingly, when averaging the MTEs to estimate an effect for this patient conditional on their observed covariates, we would not consider MTEs that imply values of the unobserved confounders that are incompatible with the choice of surgery decision, based on the econometric choice model used to study the treatment selection behavior. By taking account of the individual's context in this manner, the PeT effect is more personalized than conditional average treatment effects which average across all of the MTEs conditional on the observed covariates.

The PeT effect is defined for each patient in the analytical dataset as the difference in their probability of dyslipidemia remission or relapse over seven years with SG versus RYGB, conditional on the patients' baseline characteristics. These person-level treatment effects can be aggregated to report the effects of SG vs RYGB over the whole sample (i.e. the average treatment effect, ATE), and for each pre-specified subgroup of interest: age (< 65 years and ≥

© 2022 Coleman KJ et al. *JAMA Network Open.*

65 years), BMI (< 50 kg/m^2 and ≥ 50 kg/m^2), race/ethnicity (non-Hispanic Black, non-Hispanic White, Hispanic), having a history of CVD (yes, no), and being a smoker (ever smoked, never smoked).

Local Instrumental Variable (LIV) Approach. The LIV approach was carried out in two steps.

"First step: Modelling the exposure": In the first step, the choice of RYGB versus SG was modelled as a function of the RYGB PP IV, after controlling for all baseline risk-factors, indicators for 3-digit zip-code, year of surgery, and the denominator of the RYGB PP IV (a proxy for total surgeon experience) using a probit regression model. Results of the Probit model are shown in **eTable 3**, along with the results when the IV was not included. A likelihood ratio test between these two models suggests that the RYGB PP IV was significantly predictive with an F-statistic of 373 (the rule of thumb cut-off for a strong IV is 10).

Likelihood-ratio test	LR chi2(1) = 372.70
(Assumption: OnlyX nested in XandZ)	Prob > chi2 = 0.0000

The predicted propensity of the type of surgery received as a function of the RYGB PP IV and other covariates are shown in **eFigure 4**.

"Second step: Modelling the outcome": In the second step, we model our outcome (Y), which is either a three-level ordinal variable (levels categorized as "original dyslipidemia", "remission", and "relapse") or a continuous variable. For the ordinal variable, we use two separate cumulative logit models. The first logit model represents the log-odds of remission or original dyslipidemia versus relapse. The second logit model represents the log-odds of original dyslipidemia versus relapse or remission. Each of these models is operationalized using generalized estimating equations using a logit link function and exchangeable correlation structure.[16,17] The log-odds of outcomes **are** our LIV estimands.

Y ϵ (0,1,2) = ("original dyslipidemia", "remission", "relapse")

First Cumulative Model in the Second Step:

$log\left(\frac{\Pr(Y\le 1}{\Pr(Y>1)}\right) = \mathrm{a0} + \mathrm{a1} * \mathrm{PSi} + \mathrm{a2} * \mathrm{XLi} + \mathrm{a3} * \mathrm{PSi} * \mathrm{XLi} + \mathrm{a4} * \mathrm{XCi} + \mathrm{g(PSi)}$, where

PS_i = Estimated propensity to choose RYGB over SG from the first stage model

X_{Li} = Set of individual level clinical variables , and their interactions with PS_i.

X_{Ci} = Set of other controls such as year and zip code fixed effects, and surgery volume.

$g(PS_i)$ = Higher order polynomials of PS_i, as appropriate, determined by nested likelihood ratio tests.

Second Cumulative Model in the Second Step:

$$log\left(\frac{\Pr(Y \leq 0)}{\Pr(Y > 0)}\right) = \mathrm{b0} + \mathrm{b1} * \mathrm{PSi} + \mathrm{b2} * \mathrm{XLi} + \mathrm{b3} * \mathrm{PSi} * \mathrm{XLi} + \mathrm{b4} * \mathrm{XCi} + \mathrm{g(PSi)},$$

For each model, we allowed for interactions of clinical factors with the propensity score from step 1 and tested for an appropriate polynomial of the propensity score itself. Higher order polynomials of the propensity score were not significant. Several goodness-of-fit tests were run to ensure proper fit to data. Generalized estimating equation estimators were used to estimate population average effects over repeated measured for the same patient. Running two cumulative binary models is mathematically equivalent to running a generalized ordered (GO) logit model, which relaxed the proportional odds assumption of a regular ordered logit without losing track of the ordering (which the multinomial model does). We did not run the GO model directly because of the complexity of the LIV analysis involved.

<u>"Models for individual lipid values"</u>: Generalized estimating equations for a linear model with quadratic polynomial of time and first-order auto-regressive errors were used to model each of the four lipid indicators.

$$E(Y|X) = \mathrm{c0} + \mathrm{c1} * \mathrm{PSi} + \mathrm{c2} * \mathrm{XLi} + \mathrm{c3} * \mathrm{PSi} * \mathrm{XLi} + \mathrm{c4} * \mathrm{XCi} + \mathrm{g(PSi)},$$

The partial derivative of the predicted probability in each of the models with respect to the propensity score was used as an estimator of the marginal treatment effects in each model.[2] These effects were then aggregated to calculate the PeT effect for each individual in our sample at any specific time since surgery.[3] We explored these comparative effects at every 90-days from alternate forms of surgery.

From the first model, we obtained a PeT effect of treatment on the probability of the combined outcome of remission or retaining original dyslipidemia status. The negative of this effect would be the PeT effect of treatment on the probability of relapse. From the second

© 2022 Coleman KJ et al. *JAMA Network Open.*

model, we obtained a PeT effect of treatment on the probability of retaining original dyslipidemia status. Since the sum of the PeT effects on all three categories must add up to zero, we can obtain the PeT effect of the probability of remission as -(PeT(Relapse) + PeT(Original Dyslipidemia) = - (- PET(First Model) + PET(Second Model)).

Details on this method can be found in a recent JAMA Network Open publication and its supplement.[5] Technical details can be found elsewhere.[2,3] These individual level PeTs are then aggregated to obtain the population average treatment effect (ATE) and also conditional average treatment effects (CATEs) for a number of subgroups: for the RYGB recipient group giving the ATE on the treated (ATT), for the SG recipient group giving the ATE on the untreated (ATUT) and also by age (< 65 years and ≥ 65 years), BMI (< 50 kg/m^2 and ≥ 50 kg/m^2), race/ethnicity (non-Hispanic Black, non-Hispanic White, Hispanic), having a history of CVD (yes, no), and being a smoker (ever smoked, never smoked).

All ATEs were expressed in the probability scale for each category. All standard errors were calculated with non-parametric bootstrapping and allowed for clustering of individual outcomes over time. Data analyses were performed in Stata (StataCorp), version 15.1.

Standard IV Analysis with Two-Stage Residual Inclusion Approach

Standard methods for IV analysis do not follow the structural approach used under local IV analysis. Rather it uses a residual inclusion approach, where the residuals from the first stage (which is similar to the one used under LIV approach) is used as an additional covariate in the second stage.[18,19]

$R_i = (D_i - PS_i)$ = 1st Stage Residuals , where D_i = Treatment indicator

Second Stage regression for the first outcomes model:

$$log\left(\frac{\Pr(Y\leq 1)}{\Pr(Y>1)}\right) = (b0 + b1{*}D_i + b2{*}X_i + b4{*}C_i + b5{*}R_i),$$

Here, R_i acts as a proxy for unobserved confounders, thereby allowing a traditional regression-based estimator to estimate the effect of D on outcomes without confounding issues. The average effect is then represented by the incremental effects of D on the mean outcomes, calculated via recycled predictions.

© 2022 Coleman KJ et al. *JAMA Network Open.*

The second outcomes model follows the approach of the first model. For systolic and diastolic blood pressure, we used linear models. The fundamental challenge of this methods is that it is not guaranteed to produce an estimate of the true ATE as the theory of residual inclusion approach is only set up for a continuous treatment, and when applied to a binary treatment, produces an approximation for the ATE.[19] Instead of using raw-residuals, we used generalized residuals to obtain the best approximation.[19]

Standard Propensity Score Methods with Inverse Probability Weighting

Finally, we also explored methods that only account for observed confounding and ignore unobserved confounding. Among a variety of methods available for such adjustment, we chose the inverse probability weight with propensity score estimator because of its robust properties and its use in the prior literature in the context of bariatric surgery.

For this estimator, we have a new propensity score first stage model. It differs from the first-stage IV model in two ways: 1) The propensity model does not adjust for the IV, but only X and C, and 2) The model is saturated in that all potential interactions and second order polynomial of covariates are included for adjustment to generate the propensity score, which was used as a balancing score. Overfitting is not a concern in this stage. Let this balancing propensity score be denoted as BPS_i.

eFigure 5 illustrates the reduction in the standardized mean difference in each of the covariates with weighted by an inverse probability weight using BPS_i. It is important to note that this reduction is a result of direct adjustment of the distribution of covariates through the propensity score, whereas in **eFigure 3**, the reduction is natural without any explicit adjustment. For the second stage, these scores are used as follows to obtain an estimate for the average treatment effect, under no unobserved confounding:

$$\left(\sum_{i=1}^{N} \frac{D_i}{BPS_i}\right)^{-1} \cdot \sum_{i=1}^{N} \frac{D_i \cdot Y_{it}}{BPS_i} - \left(\sum_{i=1}^{N} \frac{1-D_i}{1-BPS_i}\right)^{-1} \cdot \sum_{i=1}^{N} \frac{(1-D_i) \cdot Y_{it}}{1-BPS_i}.$$

© 2022 Coleman KJ et al. *JAMA Network Open.*

eTable 1. Detailed inclusions and exclusions for the Effectiveness of Gastric Bypass vs. Gastric Sleeve for Cardiovascular Disease (ENGAGE CVD) study.

Criteria	Excluded	Remaining
All patients with a bariatric surgery		22,095
**Inclusions**		
Dyslipidemia at Surgery	8,110	13,985
Surgery year 2011-2016	3,175	10,810
**General Exclusions**		
At least 365 days of follow-up post-surgery	75	10,735
**Exclusions specific to IV analysis**		
Surgery total ≤ 30 for surgeon OR ≤100 for zip code area in 12 months before surgery	352	10,383
Instrumental Variable (IV)-based pseudo randomization not present (peripheral 20 percentile)	2,080	8,303
IV-based propensity score overlap not present	38	8,265

eFigure 1. Distribution of instrumental variable (IV) and the balance of normalized observed confounders over IV levels. Normalization based was on computing a z-score for each covariate by demeaning it and dividing by its standard deviation. A lowess smoother was used to examine the levels of z-score across IV.

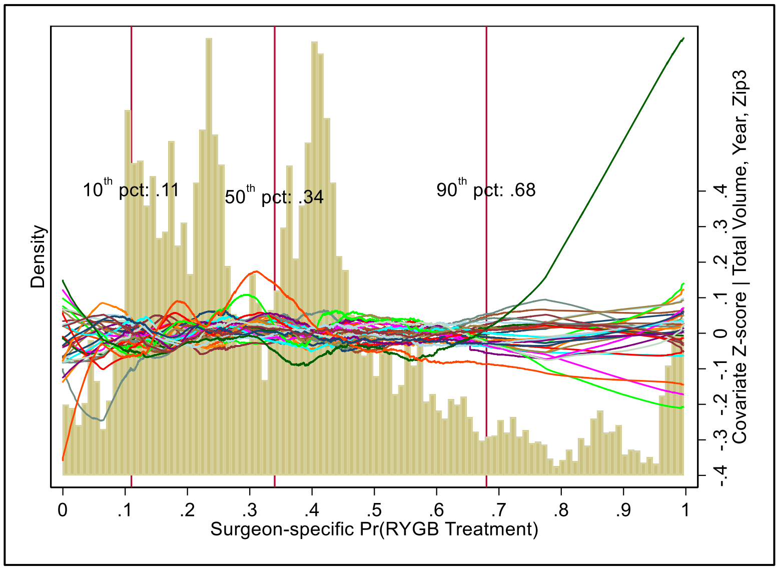

© 2022 Coleman KJ et al. *JAMA Network Open.*

eTable 2. Differences in patient characteristics for central 10 – 90 versus peripheral 20 percentile of surgeon-specific rates.

**Variable**	**Peripheral 20th percentile N = 2,080**		**Central 80th percentile N = 8,303**		**p-value**
	Mean ± SD		Mean ± SD		
Z-value for Weight Change Before Surgery	-0.09 ± 0.99		-0.01 ± 0.97		<0.001
	N	%	N	%	
Non-Hispanic Whites	836	(40.2)	3011	(36.3)	0.001
Sleep Apnea	240	(11.5)	1346	(16.2)	<0.001
CKD	365	(17.6)	1142	(13.8)	<0.001
Severe Anxiety	103	(5.0)	551	(6.6)	0.005
GERD	997	(48.0)	2711	(32.6)	<0.001
Gastric Duodenitis	149	(4.5)	266	(3.2)	<0.001

eFigure 2. Balance of covariate z-scores (a) across treatment, and (b) across IV levels (Note: These reductions in imbalance across IV median occurs naturally, without adjustment for these covariate levels).

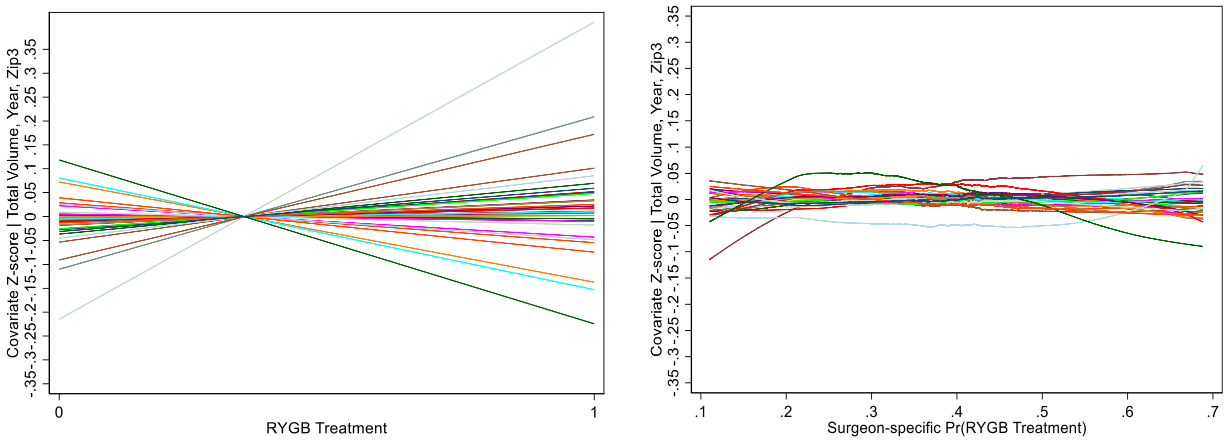

© 2022 Coleman KJ et al. *JAMA Network Open.*

eFigure 3. Standardized mean difference for each covariate across treatment versus across median of the IV (e.g. below and above the median).

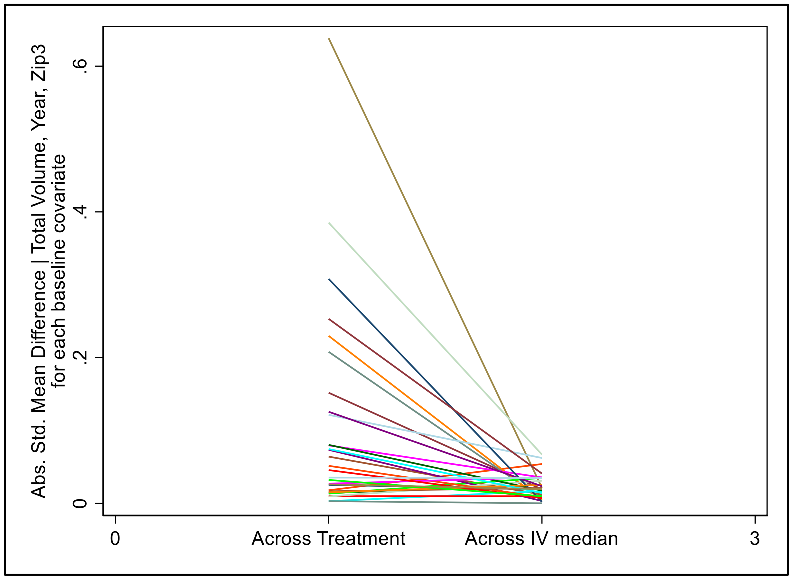

eTable 3. Factors affecting RYGB selection (First-stage IV regression).

Variable	OnlyX	XandZ
Age in years (centered at 45)	-0.008***	-0.009***
Age, centered at 45 years, squared	0.000***	0.000***
female	0.058	0.055
presurg_appattpr	0.188	0.189
z_wtch	0.018	0.008
ln_bmi	0.211	0.200
Baseline LDL	0.000	0.000
Baseline HDL	0.000	0.001
Baseline Total Cholesterol	-0.002*	-0.002**
Baseline Triglyceride	0.000	0.000
IPdays_1yrbf	-0.075	-0.084
EDvisits_1yrbf	0.005	0.003
Elixhauser	0.004*	0.004*
eversmoker	-0.037	-0.025
diabetes_atsurg	0.750***	0.772***
non_hisp_white	-0.062	-0.083
non_hisp_black	-0.249**	-0.248**

© 2022 Coleman KJ et al. *JAMA Network Open.*

hispanic_ind	-0.038	-0.049
bmi50	0.048	0.035
aspirin_1yr	0.141*	0.104
aspirin_3mo	0.027	0.098
nsaids_1yr	0.021	0.015
nsaids_3mo	-0.064	-0.057
cirrhosis	-0.105	-0.056
sleep_apnea	0.046	0.042
ckd_before	0.001	0.003
serious_mental	0.169*	0.187**
severe_anxiety	0.092	0.100
mild_mod_anxiety	0.024	0.031
GERD_2yr	0.193***	0.181***
Gastritis_duodenitis_2yr	-0.860***	-0.837***
Dyspepsia_2yr	0.150*	0.175*
Hiatal Hernia	0.358***	0.376***
Ln(Surgeon total caseload in previous year)	-0.100**	-0.124***
Ln(3-digit zipcode level total caseload in previous year)	-0.192	5.841*
Year 2012	0.009	0.155***
Year 2013	0.013	0.239***
Year 2014	-0.048	0.162**
Year 2015	-0.174***	0.100
Year 2016	-0.080	0.197***
3-digit zipcode indicator		
902	-0.143	0.635*
903	-0.527	13.198*
905	-1.009	18.864*
906	-0.137	2.058*
907	-0.240	1.682*
908	-0.513	6.586*
902	-0.143	0.635*
910	-0.713	16.861*
911	-0.870	22.834*
912	-0.725	25.193*
913	-0.293	3.508*

© 2022 Coleman KJ et al. *JAMA Network Open.*

914	-0.608	16.132*
915	-1.049	26.693*
916	-0.538	18.608*
917	0.205	-3.106*
918	-1.178	25.280*
919	0.085	7.469*
920	-0.021	7.749*
921	0.037	8.732*
922	0.034	10.391*
923	0.509	-6.268*
924	0.242	5.291*
925	0.427	-2.595*
926	-0.318	16.423*
927	-0.513	20.726*
928	0.002	5.965*
930	-0.218	11.548*
932	-1.394	18.701*
933	-0.572	3.860*
935	(omitted)	(omitted)
sg_rygbrate		2.449***
Constant	0.983	-58.061*
N	8625	8625

© 2022 Coleman KJ et al. *JAMA Network Open.*

eFigure 4. Distribution of IV-propensity score by treatment.

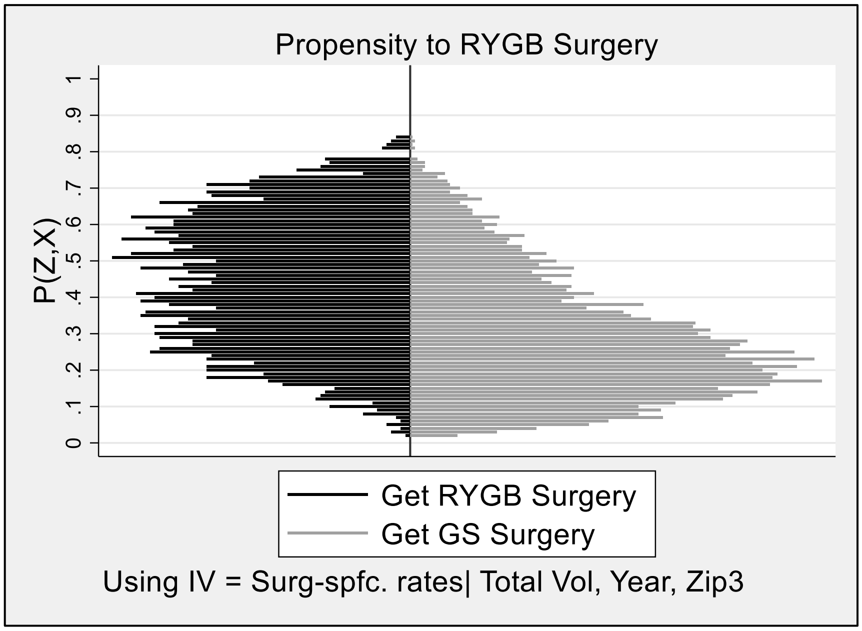

eFigure 5. Standardized mean difference for each covariate across treatment with and without propensity score-based weighting. (Note: Compared to the imbalance reduction across IV-levels, this reduction of imbalance is artificial as it is achieved by directly adjusting for the distribution of covariates).

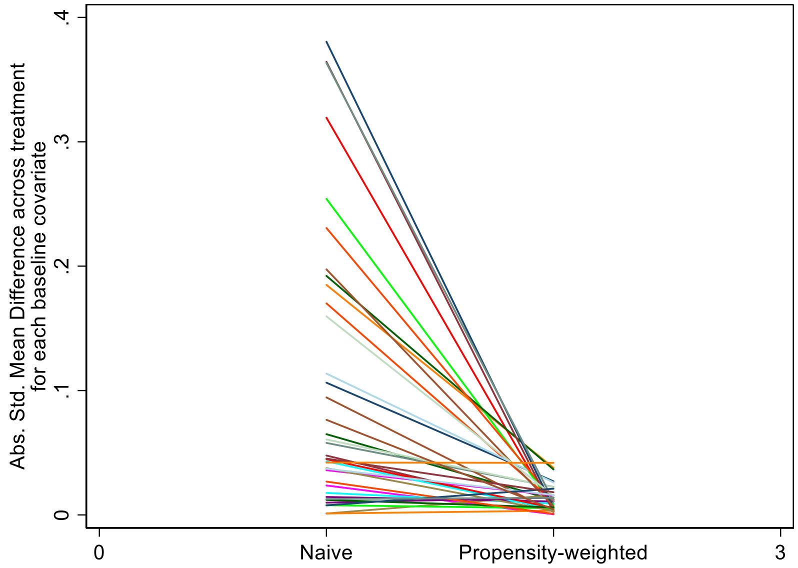

© 2022 Coleman KJ et al. *JAMA Network Open.*

eFigure 6. Trends in lipid levels with interpolation and raw data without interpolation.

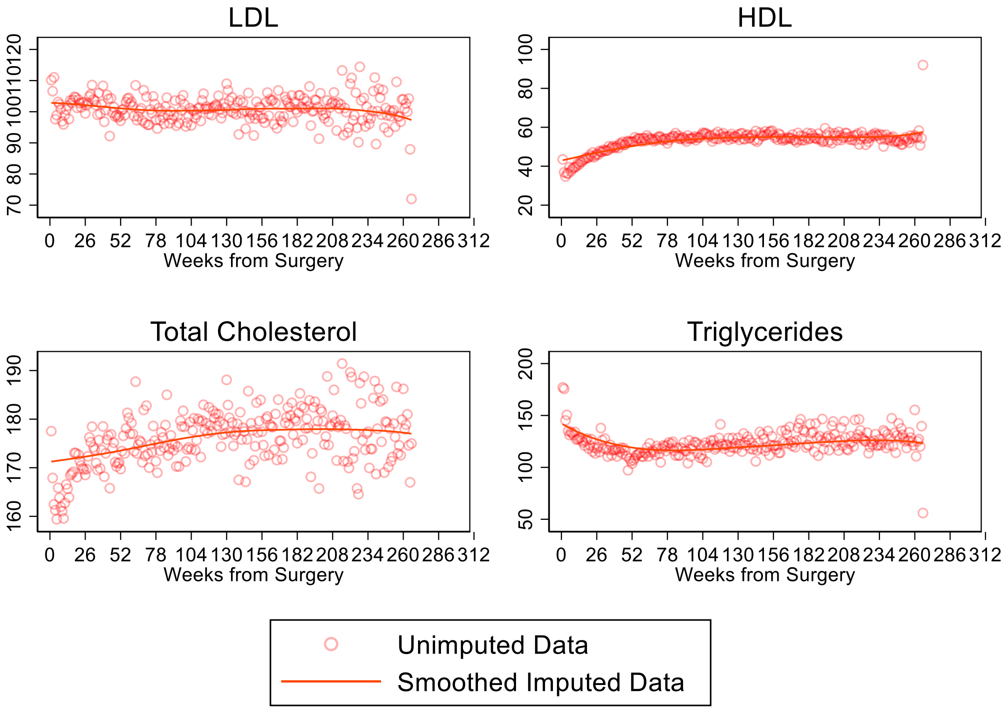

Cholesterol levels were measured at different intervals for different patients. Although, on average, the number of days between measurements was about 250 days for each of the lipid levels, all lipid levels were not always measured on the same day. We created complete data on all four lipid levels for any day on which at least one of them were measured. We achieve this by linearly interpolating each lipid level for days with missing values bookended by two corresponding measured values. No extrapolation was done for missing lipid levels. **eFigure 6** shows that the smoothed values with interpolations did not show any systematically different patterns as the measured data for any of the lipid levels. After interpolations, the average number of days between measurements was about 30 days for each of the lipid levels.

© 2022 Coleman KJ et al. *JAMA Network Open.*

eTable 4. Lipid levels for vertical sleeve gastrectomy (VSG) and Roux-en-Y Gastric Bypass (RYGB). Data are presented at baseline and in each year of follow-up for: (a) unadjusted mean levels and (b) the average difference in adjusted mean values between RYGB and VSG with 95% confidence intervals. Confidence intervals not overlapping 0 are considered statistically significant. Data are point estimates for **Figure 3** in the main manuscript.

(a) Unadjusted Mean Values									(b) Average Difference in Adjusted Mean Values (95% CI) RYGB - VSG			
	RYGB				VSG							
Days	HDL	LDL	TC	Tri	HDL	LDL	TC	Tri	HDL	LDL	TC	Tri
0	43.0	104.0	169.9	163.4	43.6	111.3	178.6	154.4	0	0	0	0
365	49.2	90.3	160.2	115.7	50.5	108.1	180.5	121.3	0.64 (-1.39, 2.66)	-16.06 (-21.91, -10.21)	-15.73 (-22.55, -8.91)	-15.54 (-27.94, -3.13)
730	53.9	89.3	163.0	112.1	54.1	107.6	183.2	119.7	0.91 (-1.24, 3.06)	-13.3 (-19.43, -7.17)	-10.76 (-17.83, -3.69)	-17.32 (-30.26, -4.37)
1095	55.4	90.1	165.4	116.1	54.8	107.5	184.3	123.8	0.23 (-1.95, 2.41)	-12.92 (-19.28, -6.55)	-10.29 (-17.53, -3.06)	-15.34 (-28.85, -1.82)
1460	55.3	89.9	165.6	119.9	54.7	107.2	184.9	128.2	-1.44 (-3.80, 0.92)	-14.26 (-21.76, -6.76)	-13.92 (-22.31, -5.54)	-10.36 (-26.33, 5.61)
1825	55.3	90.0	166.2	123.4	54.3	106.4	184.6	130.4	-4.18 (-7.33, -1.03)	-17.72 (-28.47, -6.96)	-21.29 (-32.84, -9.74)	-0.06 (-20.13, 20.01)

© 2022 Coleman KJ et al. *JAMA Network Open.*

eFigure 7. Average effects on dyslipidemia status by different analytic techniques: a) multivariate regression, b) propensity score inverse probability weighting (PS-IPW), c) two-stage residual inclusion IV approach, and d) LIV estimator.

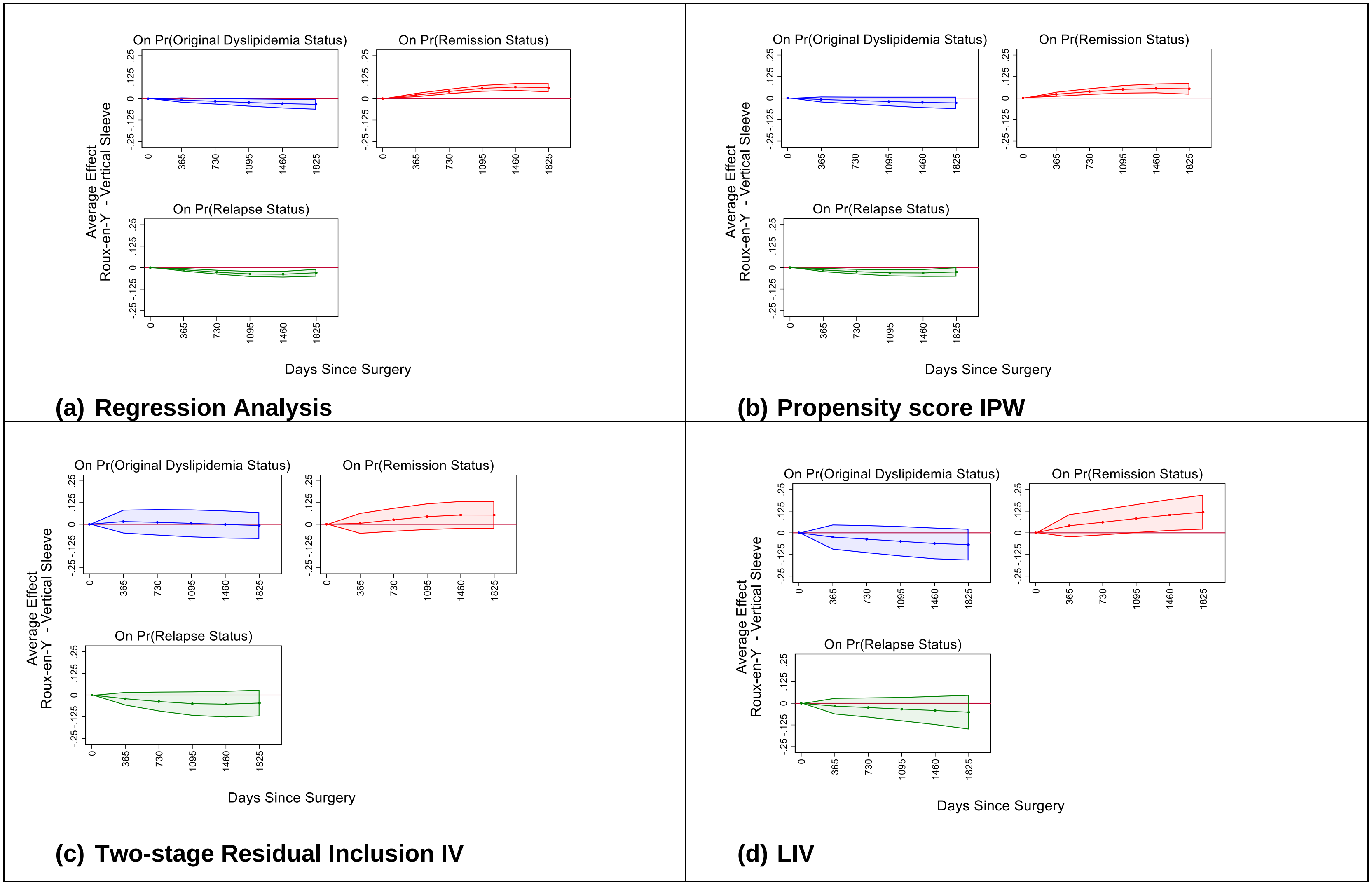

© 2022 Coleman KJ et al. *JAMA Network Open.*

eFigure 8. Average effects on lipid levels by different analytic techniques: a) multivariate regression, b) propensity score inverse probability weighting (PS-IPW), c) two-stage residual inclusion IV approach, and d) LIV estimator.

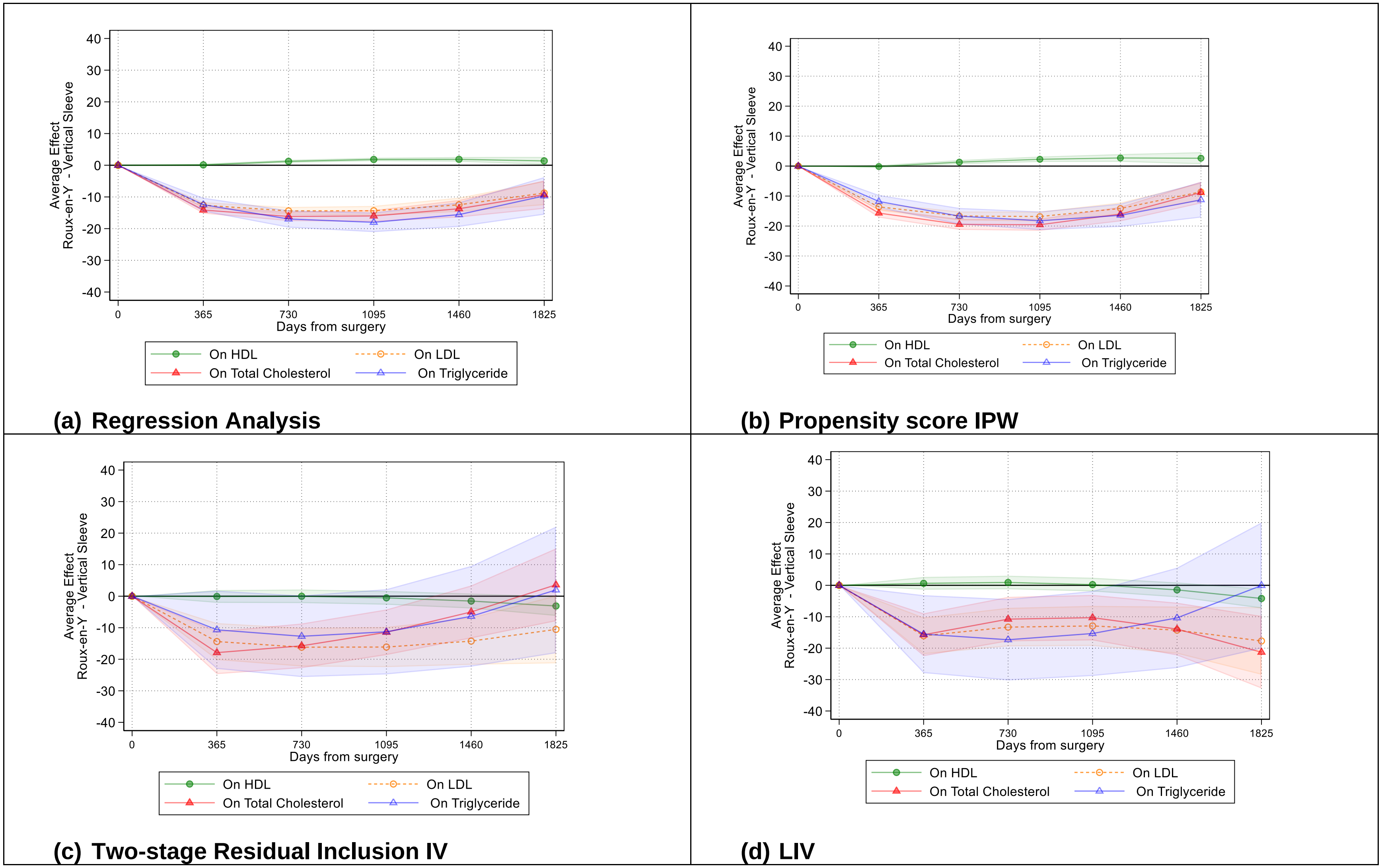

© 2022 Coleman KJ et al. *JAMA Network Open.*

eFigure 9. Comparing propensity-score-based analysis results on final sample used in LIV analysis versus on full sample with no trimming due to LIV analysis.

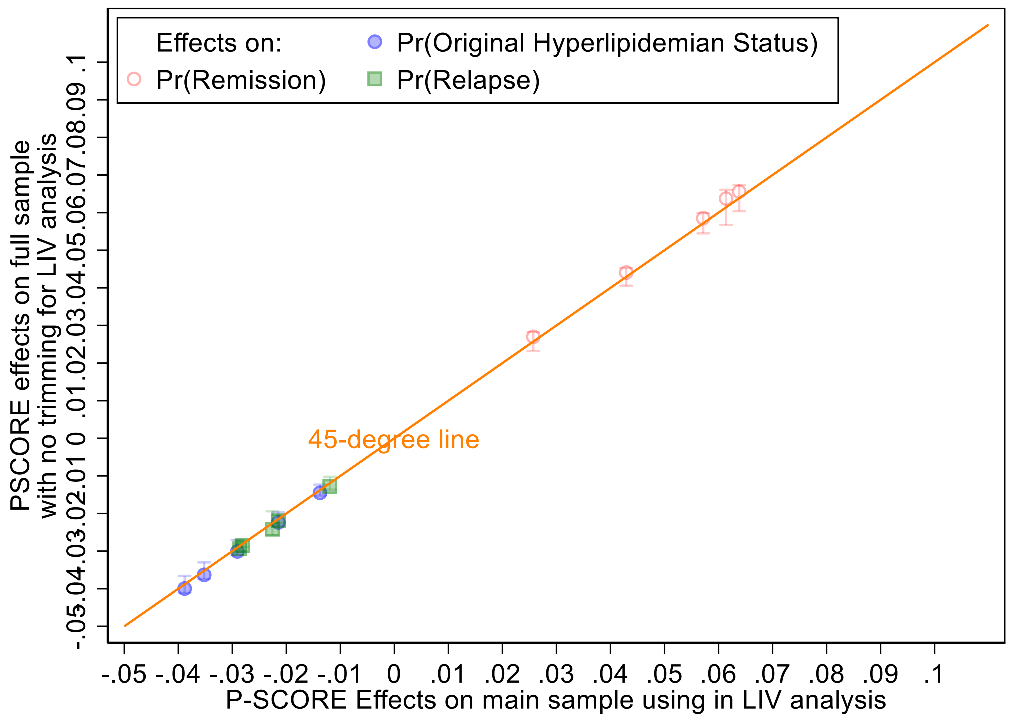

© 2022 Coleman KJ et al. *JAMA Network Open.*

eFigure 10. Hazard of attrition by bariatric surgery operation due to (a) disenrollment or (b) death.

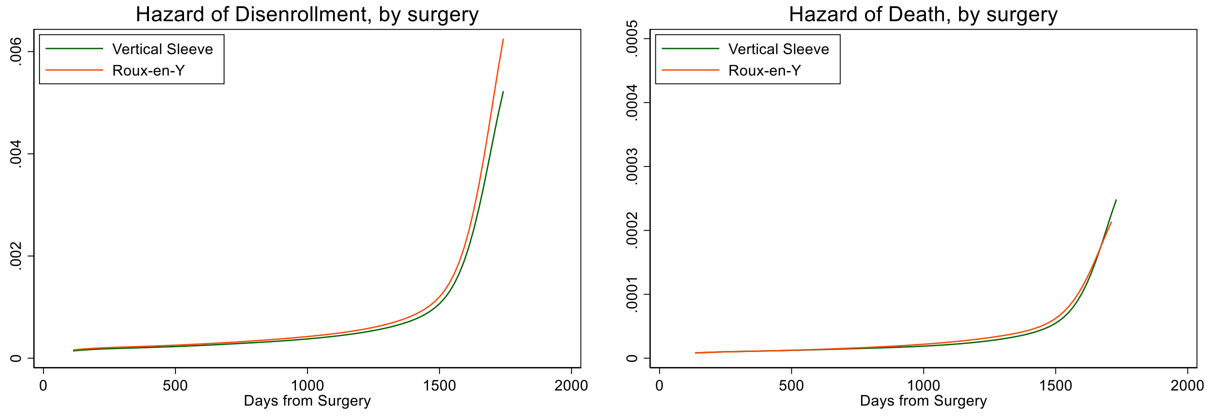

© 2022 Coleman KJ et al. *JAMA Network Open.*

eFigure 11. Adjusted proportions of patients with original dyslipidemia, remission, or relapse over 5-years for the two bariatric surgical operations by subgroups. Results are shown for overall unadjusted a) age, b) body mass index (BMI), c) race, d) Hispanic ethnicity, e) CVD at surgery, and f) smoking status at surgery.

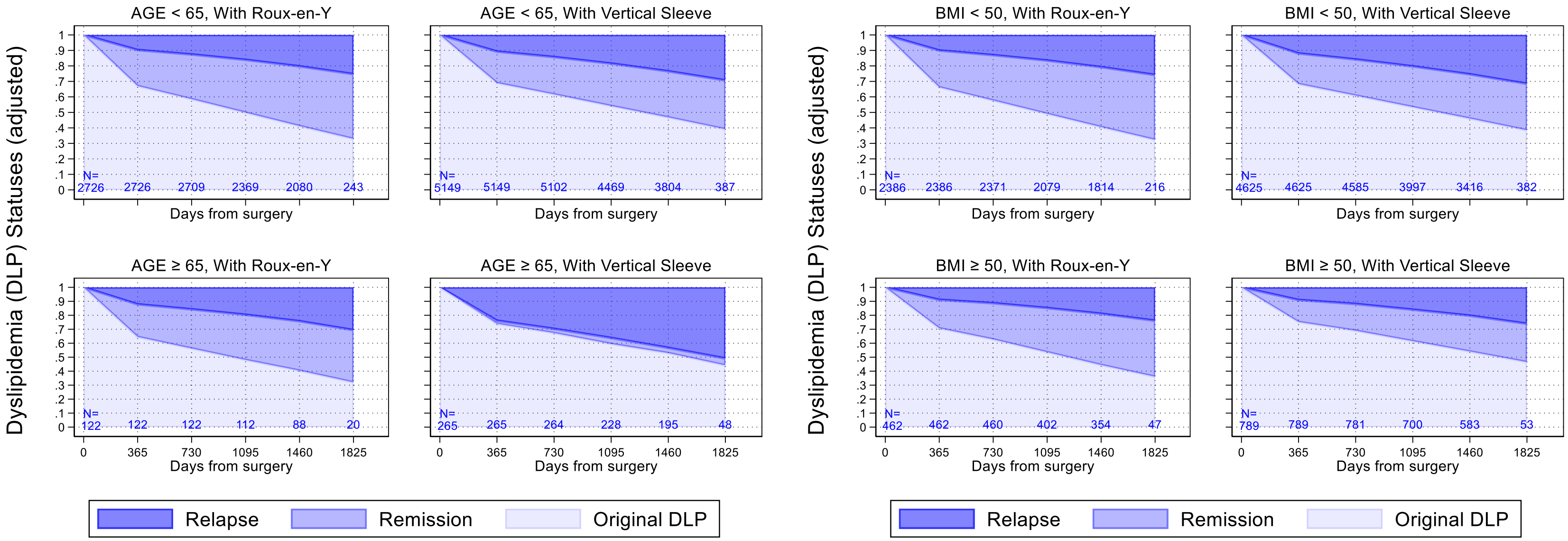

© 2022 Coleman KJ et al. *JAMA Network Open.*

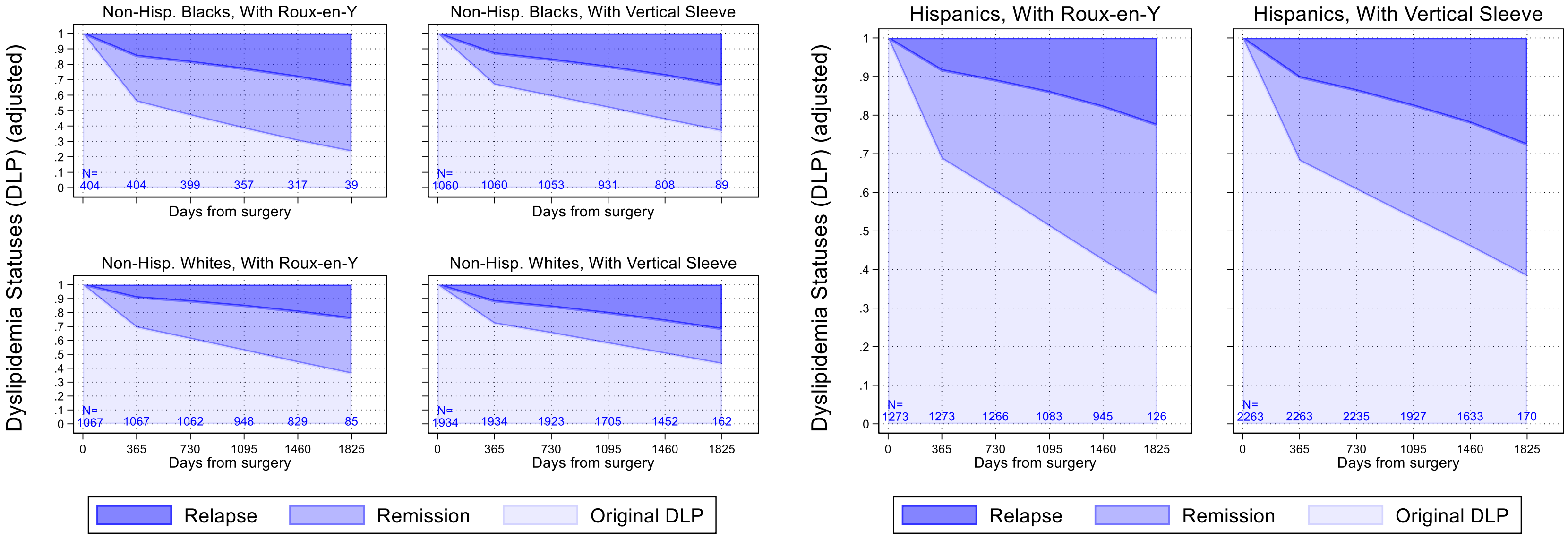

© 2022 Coleman KJ et al. *JAMA Network Open.*

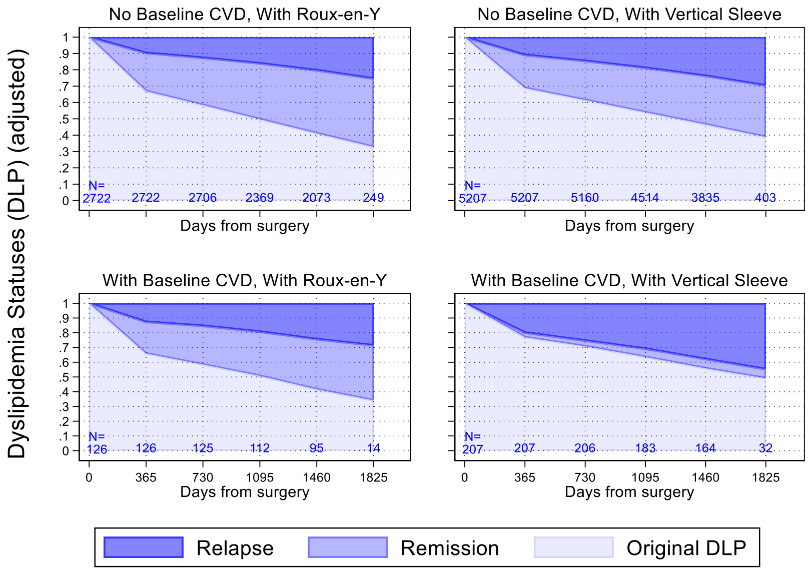

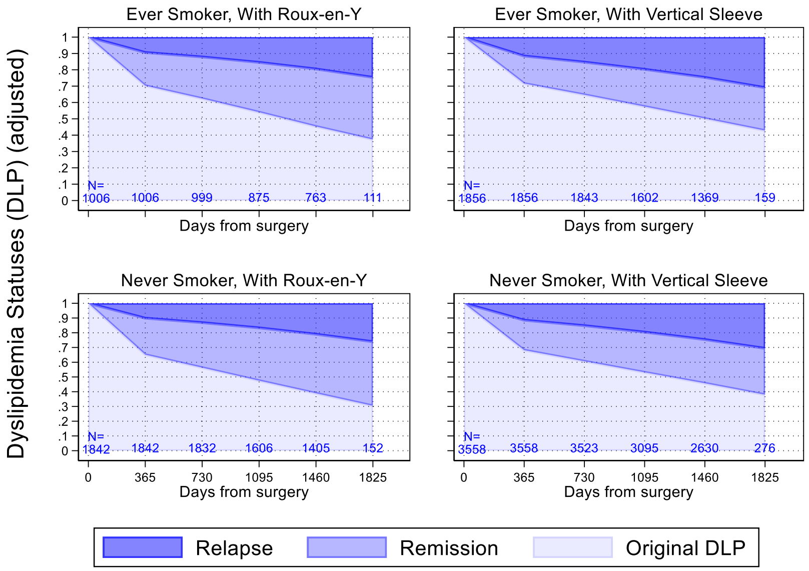

© 2022 Coleman KJ et al. *JAMA Network Open.*

eReferences

[1] Angrist J, Imbens G, Rubin D. Identification of Causal Effects Using Instrumental Variables. Journal of the American Statistical Association 1996; 91: 444-455.

[2] Basu A, Heckman J, Navarro-Lozano S, Urzua S. Use of instrumental variables in the presence of heterogeneity and self-selection: An application to treatments of breast cancer patients. Health Economics 2007; 16(11): 1133 -1157.

[3] Basu A. Estimating Person-Centered Treatment (Pet) Effects Using Instrumental Variables: An Application to Evaluating Prostate Cancer Treatments. J Appl Econ (Chichester Engl). 2014;29(4):671-691.

[4] Basu A, Gore JL. Are elderly patients with clinically localized prostate cancer overtreated? Exploring heterogeneity in survival effects. Med Care. 2015;53(1):79.

[5] Grieve R, O'Neill S, Basu A, Keele L, Rowan KM, Harris S. Grieve R, O'Neill S, Basu A, Keele L, Rowan KM, Harris S. Analysis of Benefit of Intensive Care Unit Transfer for Deteriorating Ward Patients: A Patient-Centered Approach to Clinical Evaluation. JAMA Netw Open. 2019;2(2):e187704.

[6] Baiocchi M, Small DS, Lorch S, Rosenbaum PR. Building a stronger instrument in an observational study of perinatal care for premature infants. J Am Stat Assoc. 2010;105(492):1285-1296.

[7] Neuman MD, Rosenbaum PR, Ludwig JM, Zubizarreta JR, Silber JH. Anesthesia technique, mortality, and length of stay after hip fracture surgery. JAMA. 2014;311(24):2508-2517.

[8] Keele LJ, Harris S, Grieve RD. Stronger instruments and refined covariate balance in an observational study of the effectiveness of prompt admission to the ICU in the UK. Journal of The Royal Statistical Society, Series A. 2018. https://doi.org/10.1111/rssa.12437

© 2022 Coleman KJ et al. *JAMA Network Open.*

[9] Basu A, Jones AM, Dias PR. Heterogeneity in the Impact of Type of Schooling on Adult Health and Lifestyle. Journal of Health Economics 2018; 57: 1-14.

[10] McClellan MB, Newhouse JB, McNeil BJ. 1994. Does more intensive treatment of acute myocardial infarction in the elderly reduce mortality? *Journal of the American Medical Association* **272(11)**: 859-66

[11] Newhouse J, McClellan MB. 1998. Econometrics in Outcomes Research: The Use of Instrumental Variables. *Annual Review of Public Health* **19**:17-34.

[12] Heckman JJ, Vytlacil E. 1999. Local instrumental variables and latent variable models for identifying and bounding treatment effects. *Proceedings of the National Academy of Sciences* **96(8)**: 4730-34.

[13] Heckman JJ, Vytlacil E. 2001. Local instrumental variables. In C. Hsiao, K. Morimue, and J.L. Powell (Eds.) Nonlinear Statistical Modeling: Proceedings of the Thirteenth International Symposium in Economic Theory and Econometrics: Essays in the Honor of Takeshi Amemiya, Cambridge University Press: New York, 1-46.

[14] Heckman JJ, Vytlacil E. 2005. Structural equations, treatment effects and econometric policy evaluation. *Econometrica* **73(3)**: 669-738.

[15] Swanson SA, Hernan MA. Think globally, act globally: An epidemiologist's perspective on instrumental variable estimation. Statistical Science 2014; 371-374.

[16] Papke LE, Wooldridge JM. Panel data methods for fractional response variables with an application to test pass rates. Journal of Econometrics 2008; 145: 121-133.

[17] Basu A, Manca A. Regression estimators for generic health-related quality of life and quality-adjusted life years. Medical Decision Making 2012; 32(1):59-69.

[18] Terza JV, Basu A, Rathouz PJ. Two-stage residual inclusion estimation: Addressing endogeneity in health econometric modeling. Journal of Health Economics 2008; 27(3):531-543.

[19] Basu A, Coe NB, Chapman CG. 2SLS vs 2SRI: Appropriate methods for rare outcomes and/or rare exposures. Health Economics 2018; 27(6):937-955.

© 2022 Coleman KJ et al. *JAMA Network Open.*
